# A unique poly(A) tail profile uncovers the stability and translational activation of TOP transcripts during neuronal differentiation

**DOI:** 10.1016/j.isci.2023.107511

**Published:** 2023-07-27

**Authors:** Marine Baptissart, Brian N. Papas, Ru-pin Alicia Chi, Yin Li, Dongwon Lee, Bhairavy Puviindran, Marcos Morgan

**Affiliations:** 1 Reproductive and Developmental Biology Laboratory, National Institute of Environmental Health Sciences, National Institutes of Health, Durham, NC 27709, USA; 2 Integrative Bioinformatics, Biostatistics and Computational Biology Branch, National Institute of Environmental Health Sciences, National Institutes of Health, Durham, NC 27709, USA

**Keywords:** Molecular biology, Developmental neuroscience, Cell biology

## Abstract

Cell differentiation is associated with global changes in translational activity. Here, we characterize how mRNA poly(A) tail processing supports this dynamic. We observe that decreased translation during neuronal differentiation of P19 cells correlates with the downregulation of 5′-terminal oligopyrimidine (TOP) transcripts which encode the translational machinery. Despite their downregulation, TOP transcripts remain highly stable and show increased translation as cells differentiate. Changes in TOP mRNA metabolism are reflected by their accumulation with poly(A) tails ∼60-nucleotide (nt) long. The dynamic changes in poly(A) processing can be partially recapitulated by depleting LARP1 or activating the mTOR pathway in undifferentiated cells. Although mTOR-induced accumulation of TOP mRNAs with tails ∼60-nt long does not trigger differentiation, it is associated with reduced proliferation of neuronal progenitors. We propose that while TOP mRNAs are transcriptionally silenced, their post-transcriptional regulation mediated by a specific poly(A) processing ensures an adequate supply of ribosomes to complete differentiation.

## Highlights

• A specific poly(A) tail dynamic underlies TOP mRNA metabolism during neurogenesis
• Accumulation of TOP mRNAs with tails <30-nt long reflects their high stability
• Accumulation of TOP mRNAs with tails ∼60-nt long promotes their translation
• LARP1 and the mTOR pathway shape TOP mRNA tailing in progenitor cells

## Graphical abstract

**Figure:**
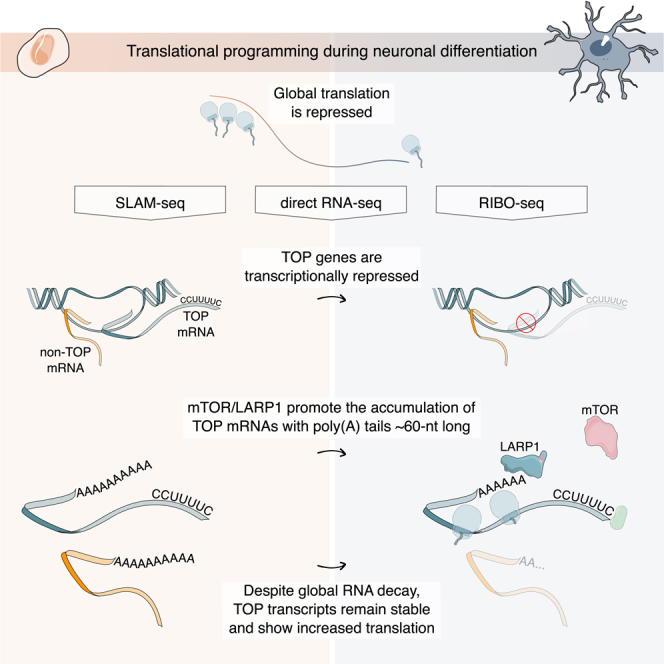

## Introduction

Stem cell differentiation is the process by which multipotent cells become more specialized while gradually losing their differentiation potential. Profound changes in global translation have emerged as a critical aspect of stem cell differentiation.^1^ This dynamic is particularly well described in neuronal systems where translation typically decreases together with ribosome biogenesis (RiBi) as stem cells differentiate into neurons.^2^^,^^3^^,^^4^ The initial availability of ribosomes is believed to provide stem cells with the capacity to synthesize a differentiated proteome, while the translational repression of differentiated cells contributes to maintain their identity.^1^ Indeed, insufficient ribosome supply impairs maturation of differentiating cells leading to developmental defects, while an excessive number of ribosomes results in stemness retention associated with cancer.^5^^,^^6^^,^^7^^,^^8^

The availability of the translational machinery relies on the regulation of ribosomal RNAs (rRNAs), as well as transcripts encoding for ribosomal proteins (RPs), ribosome biogenesis factors (RBFs), and other proteins involved in translation such as initiation and elongation factors. During differentiation, the expression of mRNAs encoding the proteins required for translation is known to be controlled at the transcriptional level. For example, the transcription of multiple RPs is controlled by myelocytomatosis oncogene (MYC), one of the four transcription factors used to obtain pluripotent stem cells from adult fibroblasts.^9^^,^^10^ However, the post-transcriptional mechanisms regulating the translational machinery during the process of differentiation remain poorly characterized.

In response to nutrient availability, the coordinated translational activation of mRNAs encoding for the translational machinery has been attributed to the 5′-terminal oligopyrimidine (TOP) motif, a stretch of approximately 4–15 pyrimidines found at the 5′ end of most transcripts encoding translation factors.^11^^,^^12^ The La-related protein 1 (LARP1) directly binds the TOP motif —and other pyrimidine-enriched sequences present in the 5′ UTR— to act as a translational switch upon phosphorylation by the mTOR complex.^13^^,^^14^ More recently, LARP1 was proposed to interact and shape the poly(A) tail of TOP mRNAs preventing their decay.^13^^,^^15^^,^^16^^,^^17^^,^^18^^,^^19^

mRNA 3′ end metabolism is a critical component of post-transcriptional regulation.^20^ In mammals, a ∼250 nucleotide (nt) long poly(A) tail is added co-transcriptionally to the 3′ end of the mRNA in the nucleus.^21^ Once released into the cytoplasm, the poly(A) tail remains coated by poly(A) binding proteins (PABPs), each covering approximately 30-nts of the tail. The presence of multiple PABPs on poly(A) tails longer than 60-nts protects the transcripts from decay and promotes their translational activation by interacting with the translation initiation complex.^22^ In contrast, the absence of PABPs on tails shorter than 30-nts facilitates transcript decay.^23^^,^^24^ In mammals, most transcripts are bound by two or more PABPs resulting in an asymmetric poly(A) tail distribution peaking at ∼60-nts, with a high frequency of tails 120-nt long.^25^^,^^26^

While poly(A) tail processing plays a central role in transcript metabolism, a correlation between poly(A) tail length and mRNA stabilization or translation has only been clearly established during germ cell differentiation and early embryogenesis. Initial findings demonstrated that the poly(A) tail length controls the translation of some transcripts during spermiogenesis or oocyte activation.^27^^,^^28^^,^^29^ Yet, the extent of poly(A) processing at the transcriptomic level remained elusive. More recently, we and others have used high-throughput methods to perform global poly(A) tail profiling and showed that poly(A) tail length defines the stability and translational activity of the whole transcriptome during gametogenesis and early development.^26^^,^^30^^,^^31^^,^^32^^,^^33^^,^^34^ Although transcriptome-wide studies of somatic cells in homeostasis found no correlation between poly(A) tail and transcript metabolism,^25^^,^^35^ several genetic studies confirmed the importance of poly(A) tail processing for somatic cell differentiation.^36^^,^^37^^,^^38^ Thus, the role of poly(A) tail length metabolism in defining the differentiating transcriptome remains to be established.

In this manuscript, we hypothesize that the translational dynamic of stem cells during neuronal differentiation is post-transcriptionally regulated through differential poly(A) tail-length processing of transcripts associated with ribosome biogenesis and translation. Using P19 pluripotent embryonic carcinoma stem cells, we show that a decrease in translational activity associated with neuronal differentiation is coupled with the downregulation of transcripts containing the TOP motif. During differentiation, TOP transcripts are repressed transcriptionally, but remain highly stable when compared to the rest of the transcriptome; this specific dynamic is associated with unique changes in their poly(A) tail length profile. While TOP mRNAs accumulate with <30-nt long poly(A) tails late in differentiation as a result of their high stability, their specific accumulation with tails ∼60-nt long from early differentiation promote their translational activation. Maintaining TOP transcripts highly stable and translationally active might be required to sustain sufficient levels of ribosome biogenesis for the cells to complete differentiation. In undifferentiated cells, the activation of mTOR pathway and the presence of its effector LARP1 can partially recapitulate the specific poly(A) tail processing of TOP mRNAs observed during neurogenesis. Although we found that the poly(A) tail processing of TOP mRNA tails is not sufficient to drive differentiation, our data support a model in which it promotes progenitor cell proliferation to ultimately define the number of differentiated neurons.

## Results

### Reduced translational activity during neuronal differentiation is associated with TOP transcripts downregulation

**Figure 1:**
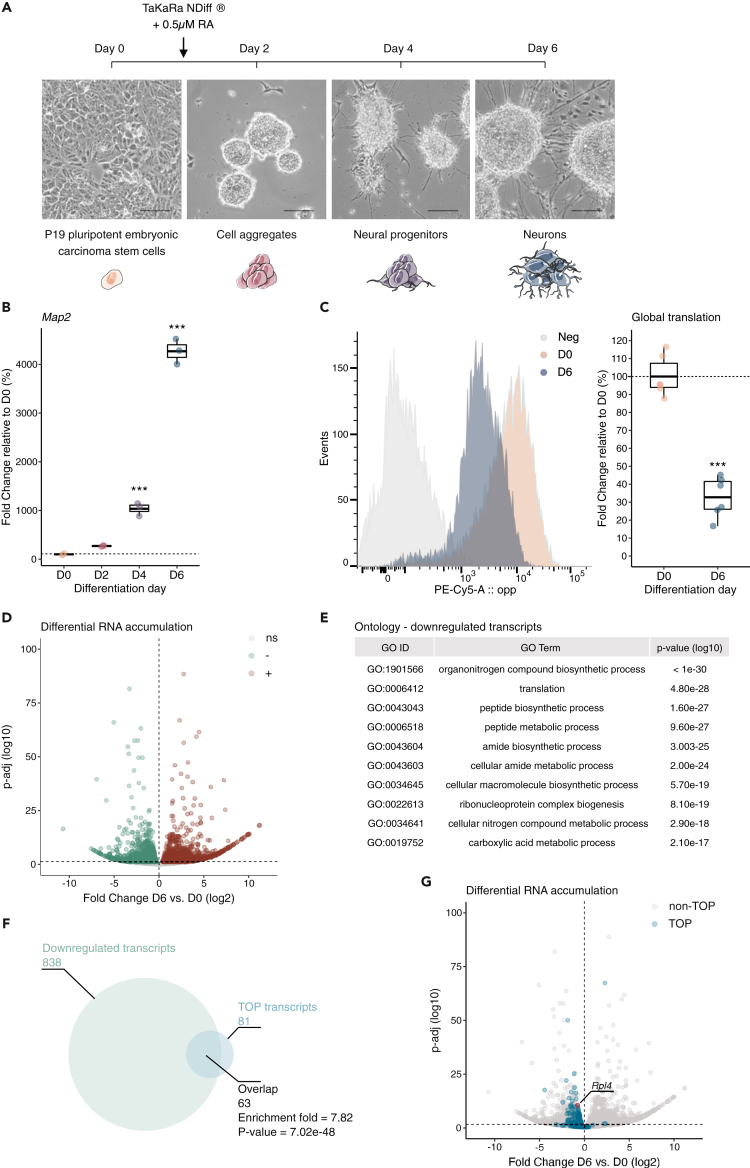
Reduced translational activity during neuronal differentiation is associated with the downregulation of TOP transcripts (A) Representative micrographs of P19 cells before differentiation (day 0, D0), and at 2 (D2), 4 (D4), and 6 (D6) days of differentiation. Scale bars: 100 μm. Schematic adaptation of “Neural cells” (composition and colors) from Servier Medical Art by Servier, licensed under a Creative Commons Attribution 3.0 Unported License (https://creativecommons.org/licenses/by/3.0/). (B) Boxplot of the relative accumulation of *Map2* transcripts before (D0) and during differentiation (D2, D4, and D6) measured by qPCR. n = 3 biological replicates. Data are expressed relative to the D0 condition. Each dot represents one replicate, the center values show the means, the boxes indicate the first and third quartiles and the bars indicate the 10^th^ and 90^th^ percentiles. Ordinary one-way analysis of variance (ANOVA), Dunnett’s multiple comparisons test. ∗∗∗p < 0.001. (C) Representative flow cytometry histogram (left panel) and quantification (right panel) of global protein synthesis measured by O-propargyl-puromycin (OPP) incorporation in cells before differentiation (D0) and after 6 days of differentiation (D6). Negative control (Neg) shows the background signal in the absence of OPP. n = 6 biological replicates. Data are expressed relative to the corresponding D0 condition. Each dot represents one replicate, the center values show the means, the boxes indicate the first and third quartiles and the bars the 10^th^ and 90^th^ percentiles. Two-tailed, two sample Student’s *t* test comparing D6 to D0. ∗∗∗p < 0.001. (D) Volcano plot of differential transcript accumulation in differentiated cells (D6) relative to undifferentiated cells (D0). Downregulated and upregulated transcripts are highlighted in green and brown, respectively. n = 3 biological replicates. Wald test corrected by Benjamini and Hochberg for multiple testing. Significance threshold q-val <0.05. (E) Table showing the 10 most enriched gene ontology terms in significantly downregulated transcripts during differentiation (from D0 to D6). Fisher’s exact test. (F) Venn diagram showing downregulated transcripts (from D0 to D6) in light green, and their intersection with TOP mRNAs in blue. Overrepresentation analysis was performed using a hypergeometric test. (G) Volcano plot shown in (D), highlighting TOP mRNAs in blue and *Rpl4* (contig ENSMUST00000034966) in pink. See also Tables S1, S2, and S3.

Stem cell differentiation is characterized by drastic changes in global protein synthesis.^1^ However, the mechanisms regulating translation during differentiation remain poorly understood. To determine how the transcriptome shapes the translational activity of differentiating cells, we employed the extensively characterized P19 model of neurogenesis, which is amenable for molecular analyses.^39^ Differentiation of the P19 cells was induced by addition of NDiff 227 medium supplemented with retinoic acid. Neurogenesis was validated morphologically by the aggregation of cells at day 2 (D2) and the presence of neuronal projections at day 6 (D6) (Figure 1A). At the molecular level, the progressive accumulation of the *microtubule-associated protein 2* (*Map2*) transcript, a marker of mature neurons, confirmed the proper neuronal differentiation of P19 cells (Figure 1B). To evaluate the levels of protein synthesis during P19 neuronal differentiation, we metabolically labeled nascent peptides and quantified their abundance by flow cytometry. Consistent with other stem cell differentiation systems,^2^^,^^3^ we found that global translation dramatically decreases as the cells commit to the neuronal lineage (Figure 1C).

Global translation can be directly influenced by the number of functional ribosomes present in the cell.^40^^,^^41^ To understand whether the decrease in global translation during neuronal differentiation is associated with changes in transcripts encoding for the translational machinery, we performed direct RNA sequencing before (D0) and after differentiation (D6). Differential RNA accumulation analysis identified ∼1000 downregulated transcripts and ∼1200 upregulated transcripts in differentiated cells (D6) compared to undifferentiated cells (D0) (Figure 1D and Table S1). Gene ontology analysis of the significantly upregulated transcripts showed an enrichment in biological processes associated with neurogenesis, as expected by the nature of our system (Table S2). Importantly, the most enriched ontology terms associated with downregulated transcripts were related to translational processes (Figures 1E and Table S2). A closer inspection at the transcript level revealed that this functional signature was driven by an enrichment for TOP transcripts, including multiple RP mRNAs encoding the small and large ribosomal subunits (Figures 1F, 1G, and Table S3). These data suggest that the downregulation of TOP transcripts contributes to a reduction in ribosome content and protein synthesis during neuronal differentiation.

### TOP transcripts remain highly stable through differentiation despite global mRNA decay

**Figure 2:**
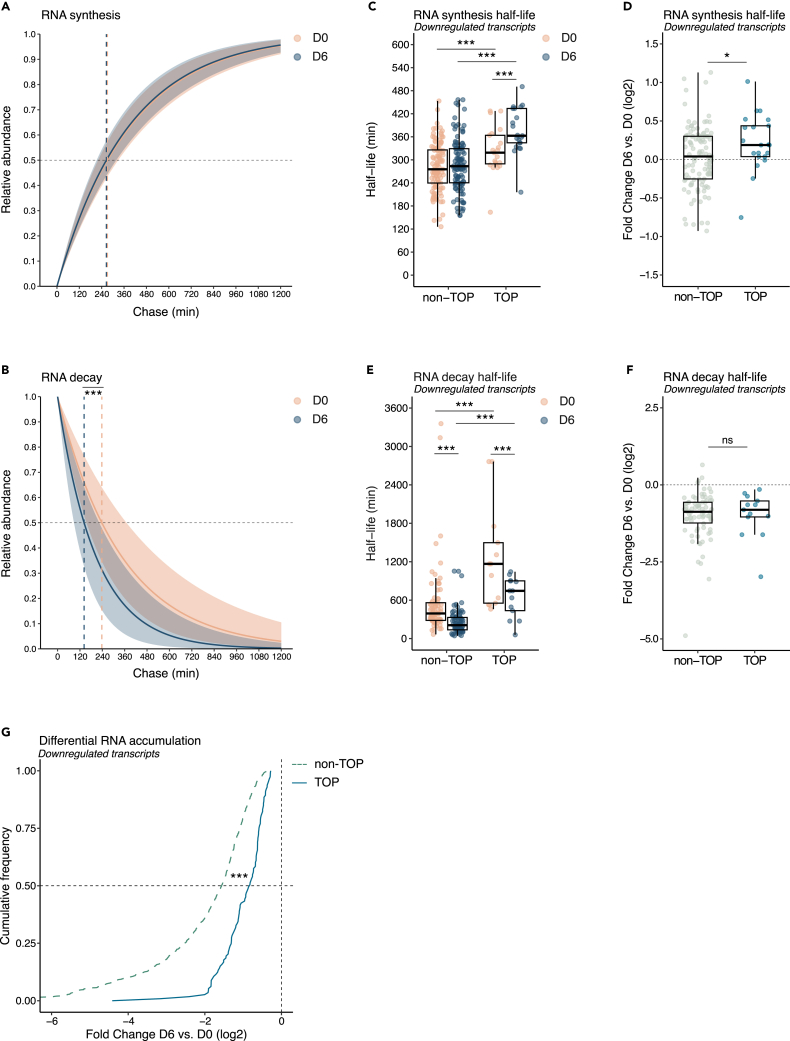
TOP transcripts remain highly stable through differentiation despite global mRNA decay (A) Plot of RNA synthesis rates for the whole transcriptome of P19 cells before differentiation (D0, orange) and after 6 days of differentiation (D6, blue). The shaded area represents the range between the first and third quartiles. Median half-lives are indicated by dashed lines. (B) Plot as in (A) for RNA decay rate. Wilcoxon signed-rank test for unpaired samples comparing D6 to D0. ∗∗∗p < 0.001. (C) Boxplot of synthesis half-life for downregulated transcripts before differentiation (D0, orange) and after 6 days of differentiation (D6, blue) split between non-TOP and TOP transcripts. Two-tailed paired Student’s *t* test comparing D6 to D0. Two-tailed, two sample Student’s *t* test comparing non-TOP to TOP transcripts. ∗∗∗p < 0.001. (D) Boxplot showing the fold change of synthesis half-life during differentiation (D6 vs. D0) for downregulated transcripts split between non-TOP and TOP transcripts. Two-tailed, two sample Student’s *t* test comparing non-TOP to TOP transcripts. ∗p < 0.05. (E) Boxplot as in (C) for decay half-life. Wilcoxon signed-rank test for paired samples comparing D6 to D0. Wilcoxon signed-rank test for unpaired samples comparing non-TOP to TOP transcripts. ∗∗∗p < 0.001. (F) Boxplot as in (D) for decay half-life. Wilcoxon signed-rank test for unpaired samples comparing non-TOP to TOP transcripts. ns, non-significant. (G) Cumulative frequency plot comparing non-TOP (dashed line, green) and TOP (solid line, blue) downregulated transcripts according to their differential accumulation before (D0) and after P19 differentiation (D6). Kolmogorov-Smirnov test, two-sided, comparing non-TOP to TOP transcripts. ∗∗∗p < 0.001. For all plots, n = 3 biological replicates. For boxplots, each dot represents one transcript, the center values show the means, the boxes indicate the first and third quartiles and the bars indicate the 10^th^ and 90^th^ percentiles. See also Figure S1.

Differential transcript accumulation could be driven by changes in mRNA production, i.e., transcriptional regulation, or by changes in mRNA decay controlled post-transcriptionally. To better understand the mechanisms underlying the transcriptomic changes associated with neuronal differentiation, we measured mRNA synthesis and decay rates using thiol SH-Linked alkylation for the metabolic sequencing of RNA (SLAM-seq).^42^ No change in global mRNA synthesis rates was observed during differentiation (Figures 2A and S1A). Analysis of decay rates, however, revealed a global reduction in mRNA half-life when comparing differentiated to undifferentiated cells (Figures 2B and S1B). The overall degradation of the transcriptome which might contribute to the reduced translational activity in differentiated cells is indicative of a strong post-transcriptional regulation of neuronal differentiation.

We next inspected the metabolism of TOP mRNAs by comparing them to other downregulated transcripts to understand the specificity of their downregulation during neuronal specification. At D0, TOP transcripts showed significantly lower synthesis rates compared to other downregulated transcripts (Figure 2C). During differentiation, their transcription was further repressed, whereas that of other downregulated transcripts was maintained (Figures 2C and 2D). The specific repression of TOP mRNA transcription was associated with a downregulation of MYC transcriptional signature (Figure S1C and Table S4).

Analysis of decay rates showed that TOP mRNAs degrade faster during differentiation with an acceleration equivalent to that of other downregulated transcripts (Figures 2E and 2F). However, their stability, already high at D0 (∼20 h), remains relatively high at D6 when compared to other transcripts (2X) (Figure 2E). The absolute stability of TOP mRNAs over differentiation might thus limit the extent of their downregulation associated with their transcriptional repression. Consistently, the downregulation of TOP transcript was less pronounced than that of non-TOP mRNAs (Figure 2G). Taken together, these results suggest that undifferentiated cells are responsible for producing a pool of TOP transcripts. As the cells differentiate, the transcription of TOP mRNAs is specifically repressed, but their absolute stability allows them to remain in relatively high abundance throughout differentiation when compared to other downregulated transcripts with a higher turnover.

### TOP transcripts show specific poly(A) tail-length processing during differentiation

**Figure 3:**
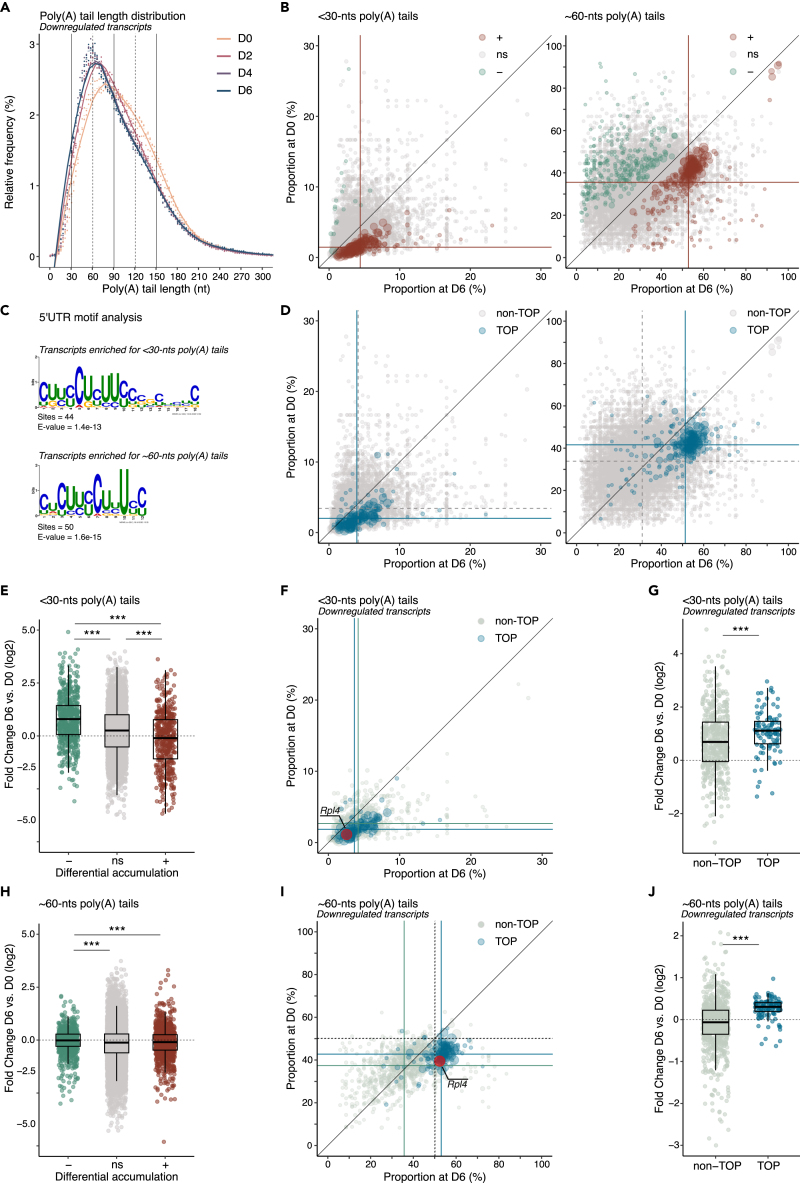
TOP transcripts show specific poly(A) tail length processing during differentiation (A) Relative frequency plot of poly(A) tail length for downregulated transcripts before differentiation (D0, orange) and at day 2 (D2, pink), 4 (D4, purple), and 6 (D6, blue) of differentiation. Dots indicate values for individual replicates and the bars indicate the relative mean frequency for each poly(A) tail length. (B) Scatterplot showing for each transcript the proportion of mRNA molecules with <30 (left panel) or ∼60 (right panel) nucleotide (nt) long poly(A) tails before differentiation (D0) and after 6 days (D6) of differentiation. Transcripts significantly decreasing (−) or increasing (+) their accumulation with <30-nt or ∼60-nt long tails during differentiation are shown in green or brown, respectively. Student’s *t* test, two-tailed comparing D6 to D0. Significance threshold p < 0.05. ns: non-significant. (C) Consensus sequences for the most enriched motif within the 5′UTR of transcripts accumulating with <30- or ∼60-nt long tails during differentiation (from D0 to D6). E-values represent the log likelihood ratio (LLR) of the occurrences of the motif corrected for multiple testing. (D) Scatterplot shown in (B) with TOP transcripts highlighted in blue. (E) Boxplot showing the fold change in the proportion of transcripts with <30-nt long tails during differentiation (from D0 to D6). Individual transcripts are grouped according to their differential accumulation (D6 vs. D0; -, downregulated; ns, no significant changes; +, upregulated). Games-Howell analysis of unequal variance. ∗∗∗p < 0.001. (F) Scatterplot of downregulated transcripts shown in (D, left), with non-TOP and TOP mRNAs indicated in light green and blue, respectively. The TOP mRNA *Rpl4* (contig ENSMUST00000034966) is highlighted in pink. (G) Boxplot as in (E) for downregulated transcripts split between non-TOP and TOP transcripts. Wilcoxon signed-rank test for unpaired samples comparing non-TOP to TOP transcripts. ∗∗∗p < 0.001. (H) Boxplot as in (E) showing the fold change in the proportion of transcripts with ∼60-nt long tails during differentiation (from D0 to D6). Krustal-Wallis test using the Bonferroni correction for multiple testing. ∗∗∗p < 0.001. (I) Scatterplot of downregulated transcripts shown in (D, right), with non-TOP and TOP mRNAs indicated in light green and blue, respectively. The TOP mRNA *Rpl4* (contig ENSMUST00000034966) is highlighted in pink. (J) Boxplot as in (H) for downregulated transcripts split between non-TOP and TOP transcripts. Wilcoxon signed-rank test for unpaired samples comparing non-TOP to TOP transcripts. ∗∗∗p < 0.001. For all plots, n = 3 biological replicates. For scatterplots, each dot represents an individual transcript. The size of each dot is proportional to the average of the transcript relative frequency at D0 and D6. The medians of both variables are indicated by lines for each population of transcripts. The identity line is shown for reference. For boxplots, each dot represents one transcript, the center values show the medians, the boxes indicate the first and third quartiles and the bars the 10^th^ and 90^th^ percentiles. See also Figure S2, Tables S5, and S6.

Given TOP mRNA transcriptional repression and high stability through differentiation, we hypothesized that their metabolism might be particularly sensitive to post-transcriptional regulation. Changes to the poly(A) tail length are one of the major post-transcriptional modifications controlling mRNA fate.^20^ To profile the poly(A) tail length dynamic of cells prior to, and during neuronal differentiation we used Oxford Nanopore Technologies (ONT) long-read sequencing.^43^ Based on the poly(A) tail length distribution, the transcriptome of stem cells showed two main populations of mRNAs. While most transcripts had poly(A) tails ∼60-nt long, a significant proportion had extended tails reaching ∼120-nts or more (Figure S2A). Interestingly, the population of downregulated transcripts showed the most dramatic changes in poly(A) tail profiles with tails accumulating with lengths around 60-nts and below 30-nts during differentiation (Figures 3A, S2B, and S2C).

To define the nature of the mRNAs associated with this dynamic, we quantified for each transcript the percentage of reads with poly(A) tails ∼60-nt and <30-nt long in both undifferentiated (D0) and differentiated cells (D6). We identified ∼220 annotated transcripts significantly accumulating with tails ∼60-nt long and ∼160 significantly accumulating with tails shorter than 30-nts after differentiation (Figure 3B). To gain insights into the pathways controlling the differential poly(A) tail dynamic of these transcripts, we searched for common regulatory sequences in their untranslated regions. A MEME analysis revealed an enrichment for the TOP motif in the 5′UTR of transcripts accumulating with ∼60-nt and <30-nt long tails, while an expected enrichment for the poly(A) signal was detected in the 3′UTR of these transcripts (Figure 3C and Table S5). Consistently, transcripts showing changes in poly(A) tail length were enriched for TOP mRNAs (Figures 3D, S2D, S2E, and Table S6), and overrepresented in ontologies related to translational processes (Table S7).

We then asked whether the poly(A) tail dynamic was strictly specific to TOP mRNAs, or common to other downregulated transcripts. We found that the accumulation of transcripts with tails shorter than 30-nts was common to all downregulated transcripts (Figure 3E), with TOP mRNAs showing the highest accumulation (Figures 3F, 3G, S2F, and S2G). In contrast, the accumulation of downregulated transcripts with tails ∼60-nt long was driven exclusively by TOP transcripts (Figures 3H–3J, S2F, and S2G). Taken together, these findings demonstrate the unique poly(A) tail length processing of TOP transcripts during neuronal differentiation.

### The poly(A) tail dynamic of TOP mRNAs is associated with their absolute stability and translational activation during neuronal differentiation

**Figure 4:**
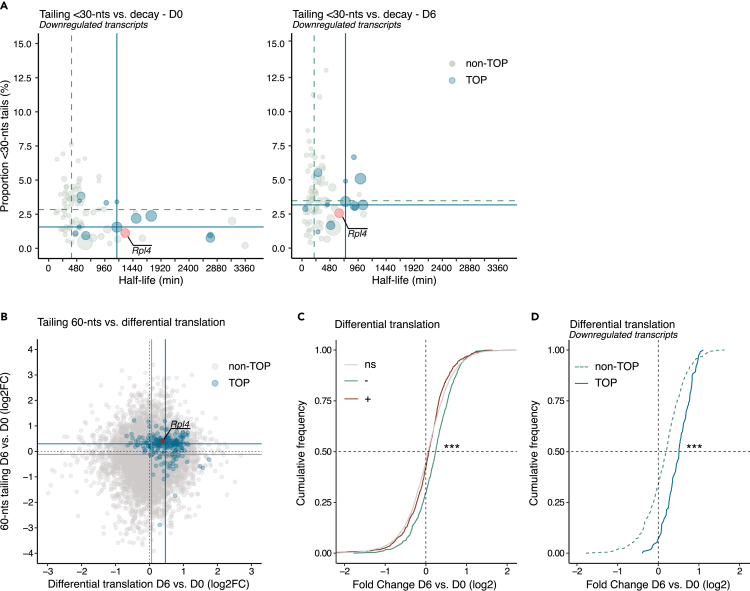
The poly(A) tail dynamic of TOP mRNAs is associated with their absolute stability and translational activation during neuronal differentiation (A) Scatterplot comparing the proportion of <30-nucleotide (nt) long poly(A) tails and decay half-life for downregulated transcripts before (D0; left) and after differentiation (D6; right). TOP and non-TOP transcripts are indicated in blue and light green, respectively. The TOP mRNA *Rpl4* (contig ENSMUST00000034966) is highlighted in pink. The size of each dot is proportional to the average of the transcript relative frequency at D0 and D6. (B) Scatterplot comparing changes in the proportion of ∼60-nt long poly(A) tails and the differential translation of transcripts during differentiation (from D0 to D6). TOP transcripts are indicated in blue. The TOP mRNA *Rpl4* (contig ENSMUST00000034966) is highlighted in pink. For both scatterplots, each dot represents an individual transcript. The medians of both variables are indicated by lines for each population of transcripts. (C) Cumulative frequency plots of relative translational change before (D0) and after differentiation (D6). The transcripts are grouped according to their differential RNA accumulation (D6 vs. D0; -, downregulated; ns, no significant changes; +, upregulated). Kolmogorov-Smirnov test, two-sided, comparing downregulated to transcripts showing no significant changes in accumulation. ∗∗∗p < 0.001. (D) Cumulative frequency plots as in (C) for downregulated transcripts split between non-TOP (dashed line, green) and TOP transcripts (solid line, blue). Kolmogorov-Smirnov test, two-sided, comparing non-TOP to TOP transcripts. ∗∗∗p < 0.001. For all plots, n = 3 biological replicates. See also Figure S3.

We next sought to determine whether the differential poly(A) dynamic of TOP mRNAs revealed specific changes to their metabolism during differentiation. Poly(A) tail shortening below 30-nts is associated with transcript decay.^24^ Globally, the transcriptome of differentiating cells behaves accordingly; accumulation of tails <30-nt long correlates with reduced half-life throughout differentiation (Figure S3A). However, a closer inspection of the downregulated transcripts shows a deviation from this pattern. Although TOP transcripts accumulate with <30-nt long tails in a higher proportion than other downregulated transcripts (Figure 3G), this was not associated with differential changes in relative stability during differentiation (Figure 2F). Instead, we observed that the absolute stability characteristic of TOP mRNAs is associated to their increased accumulation with <30-nt long tails during differentiation (Figure 4A). Together these results show that unlike the rest of the transcriptome, the <30-nts accumulation of TOP mRNAs during differentiation is not only related to their decay but also reflects their higher absolute stability.

We next sought to determine whether the accumulation of transcripts with tails ∼60-nt long was associated with translational activation of mRNAs in our system. To this end, we performed Ribo-seq before differentiation (D0) and after 6 days of differentiation (D6). The Ribo-seq protocol consists in the generation of libraries from mRNA fragments protected by the ribosome under different conditions which provide direct evidence of ribosome and mRNA engagement. A whole transcriptome analysis showed that mRNAs accumulating with tails ∼60-nt long during differentiation had increased translational activation as observed in other systems^26^^,^^27^^,^^28^ (Figure S3B). Similarly, TOP transcripts clustered with both a high proportion of 60-nt long tails and increased translational activity after neuronal differentiation (Figure 4B). The cumulative distribution of translational change between undifferentiated and differentiating conditions showed higher translational activation of downregulated mRNAs compared to upregulated and unchanged mRNAs (Figure 4C). Among downregulated transcripts, the population of TOP mRNAs showed the most pronounced changes in translational activity (Figure 4D). Of note, although the translational activity of TOP transcripts increases as cells differentiate, we did not observe any upregulation of gene ontology categories associated with translation in the differentiated cells, suggesting mostly a post-transcriptional activation of the translational machinery (Table S2). Moreover, the translational changes during differentiation were associated with the translational signatures of mTOR and LARP1, two known regulators of TOP transcripts translation (Figure S3C and Table S8). Altogether, these results show that the unique poly(A) tail profile of TOP mRNAs is associated with their absolute stability and translational activation as the cells progress through differentiation.

### mTOR and LARP1 regulation partially recapitulates the poly(A) dynamic of TOP mRNAs during neuronal differentiation

**Figure 5:**
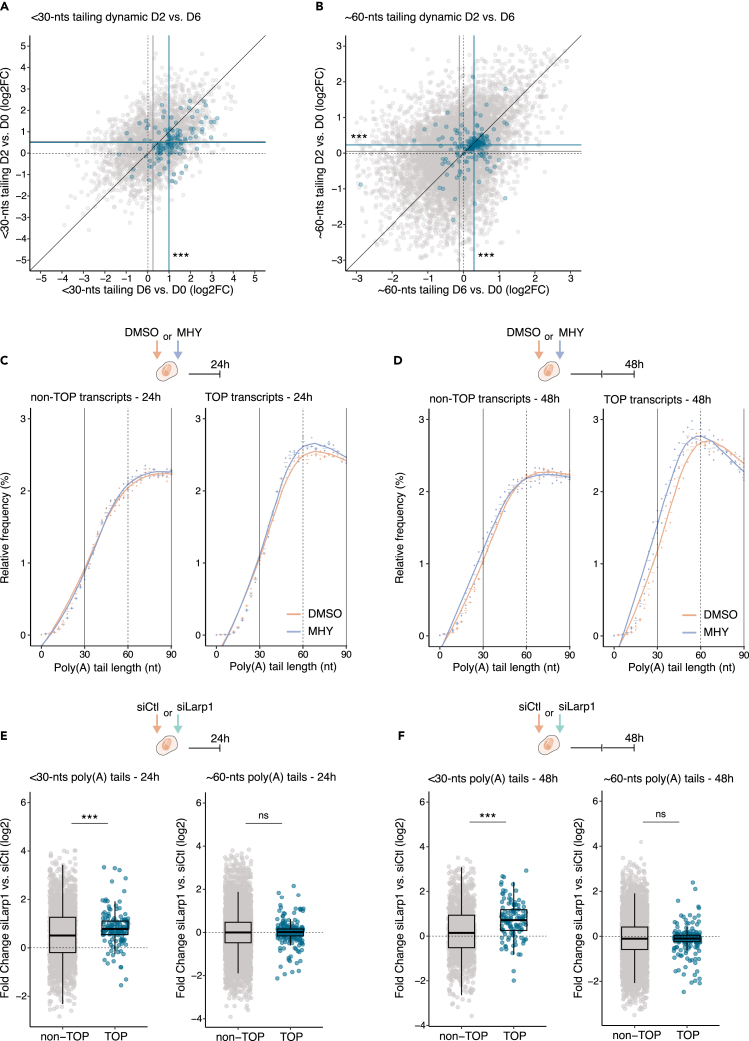
LARP1 and mTOR regulation partially recapitulates the poly(A) dynamic of TOP mRNAs during neuronal differentiation (A) Scatterplot comparing the change in proportion of <30-nucleotide (nt) long poly(A) tails after 2 days (D2) or 6 days of differentiation (D6). Transcripts encoding TOP mRNAs are indicated in blue. n = 3 biological replicates. Wilcoxon signed-rank test for unpaired samples comparing non-TOP to TOP transcripts at D2 or D6. ∗∗∗p < 0.001. (B) Scatterplot as in (A) comparing the change in proportion of ∼60-nt long poly(A) tails. n = 3 biological replicates. Wilcoxon signed-rank test for unpaired samples comparing non-TOP to TOP transcripts at D2 or D6. ∗∗∗p < 0.001. For both scatterplots, data are representative of three biological replicates per condition. Each dot represents an individual transcript. The medians of both variables are indicated by lines for each population of transcripts. (C) Relative frequency plot of poly(A) tail length for the non-TOP (left) and TOP (right) transcripts after 24 h of exposure to DMSO (orange) or MHY1485 (MHY, blue). n = 2 biological replicates. Dots indicate values for individual replicates and bars indicates the relative mean frequency for each poly(A) tail length. (D) Relative frequency plot as in (C) after 48 h of exposure to DMSO (orange) or MHY1485 (MHY, blue). n = 2 biological replicates. (E) Boxplot showing the fold change in the proportion of tails <30-nt (left) and ∼60-nt (right) long 24 h after transfection with siCtl or siLarp1. TOP mRNAs are compared to non-TOP mRNAs. n = 3 biological replicates. Wilcoxon signed-rank test for unpaired samples comparing non-TOP to TOP transcripts. ∗∗∗p < 0.001. ns, non significant. (F) Boxplot as in (E) 48 h after transfection. n = 2 biological replicates. Wilcoxon signed-rank test for unpaired samples comparing non-TOP to TOP transcripts. ∗∗∗p < 0.001. ns, non significant. For all boxplots, each dot represents an individual transcript, the center values show the medians, the boxes indicate the first and third quartiles and the bars the 10^th^ and 90^th^ percentiles. See also Figures S4 and S5.

To better understand the mechanisms regulating the dynamic of TOP mRNA poly(A) tails during differentiation, we analyzed the accumulation of tails ∼60-nt and <30-nt long after 2 days of differentiation. While most TOP mRNAs already showed accumulation with tails ∼60-nt long, we observed no accumulation of transcripts with tails <30-nt long at this time point (Figures 5A and 5B). This supports a model in which the accumulation of TOP mRNAs with ∼60-nt long tails is initially driven by an acceleration of their transition from 120- to 60-nts, while tails are protected from further shortening below 30-nts.

The mTOR pathway is known to preferentially regulate the translation of TOP mRNAs. This preference is mediated by LARP1 which binds the TOP mRNAs to act as a translational switch in response to mTOR.^11^^,^^13^ More recently, LARP1 was proposed to also shape the tails of TOP mRNAs by protecting them from deadenylation.^16^^,^^17^^,^^18^^,^^19^ Given the correlation between TOP mRNAs translational activation and their accumulation with poly(A) tails ∼60-nt long, we speculated that mTOR and LARP1 control the poly(A) tail dynamic of TOP transcripts in our system. To investigate this possibility, we first activated the mTOR pathway in undifferentiated cells using the agonist MHY1485. Western blot analysis of ribosomal protein S6 phosphorylation confirmed an increase in the mTOR activity after 24 and 48 h of exposure (Figure S4A). We then used direct RNA sequencing to profile poly(A) tail changes associated with mTOR activation. At 24 h, MHY1485 treatment induces the specific accumulation of TOP transcripts with ∼60-nt long tails, while the proportion of tails <30-nt long was initially reduced (Figures 5C, S4B, and S4D). By 48 h, the fraction of TOP mRNAs with tails ∼60-nt long was no different between control and treated cells, but the proportion of transcripts with poly(A) tails shorter than ∼30-nts increased significantly (Figures 5D, S4C, and S4E). These results show that in undifferentiated cells, mTOR activation is sufficient to recapitulate the transition of TOP mRNAs from ∼120-nt to ∼60-nt long tails, followed by their accumulation with tails shorter than 30-nts.

To understand the contribution of LARP1 to the differential poly(A) tail dynamic of TOP mRNAs, we used short interfering RNA (siRNA) to knock down *Larp1* for 24 and 48 h in undifferentiated cells. The reduction in LARP1 protein levels was confirmed by Western blot (Figure S5A); and no noticeable effects were observed on cell number or morphology (Figure S5B). Direct RNA sequencing after 24 h showed that the whole transcriptome of LARP1-depleted cells accumulated with tails <30-nt long when compared to control cells (Figures 5E and S5C). Interestingly, while TOP transcripts showed higher accumulation with <30-nt long tails compared to the rest of the transcriptome by 24 h, they were the only ones that still accumulated with <30-nt long tails after 48 h (Figures 5F and S5D). These results show that in undifferentiated cells, LARP1 prevents the widespread shortening of tails from ∼60-nts to under 30-nts. By conferring additional protection to TOP mRNAs, LARP1 may actively contribute to their accumulation with ∼60-nt long poly(A) tails, while the activation of mTOR accelerates their transition from ∼120-nt to ∼60-nt long tails. Altogether, our findings show that mTOR and LARP1 can partially recapitulate the specific poly(A) tail processing of TOP mRNAs observed during differentiation.

### The timely processing of TOP mRNA poly(A) tails is associated with the expansion of neuronal progenitors

**Figure 6:**
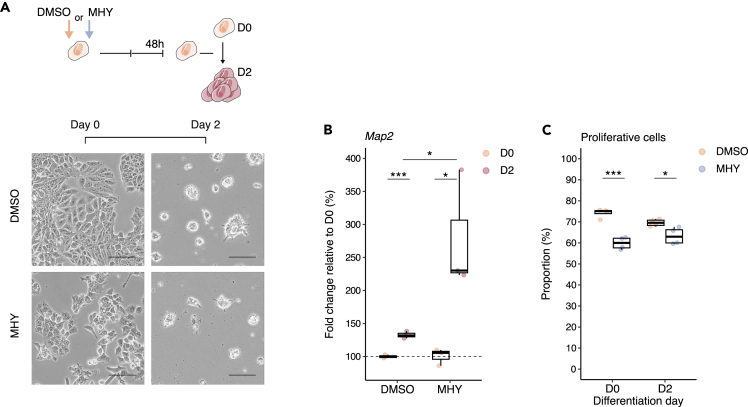
The timely processing of TOP mRNA poly(A) tails is associated with the expansion of neuronal progenitors (A) Representative micrographs of P19 cells treated with DMSO or MHY1485 (MHY), before differentiation (D0) and after two days of differentiation (D2). Scale bars: 100 μm. Schematic adaptation of “Neural cells” (composition and colors) from Servier Medical Art by Servier, licensed under a Creative Commons Attribution 3.0 Unported License (https://creativecommons.org/licenses/by/3.0/). (B) Boxplot of the relative accumulation of *Map2* transcripts before differentiation (D0) and after two days of differentiation (D2) in the presence of DMSO or MHY1485 (MHY). n = 3 biological replicates. Data are expressed relative to the D0 condition. Two-tailed, two sample Student’s *t* test, comparing D0 to D2 or DMSO to MHY1485. ∗p < 0.05; ∗∗∗p < 0.001. (C) Boxplot showing the proportion of proliferating cells before (D0) and after (D2) differentiation in the presence of DMSO or MHY148 (MHY). n = 6 biological replicates. Games-Howell analysis of unequal variance comparing D0 to D2 or DMSO to MHY1485. ∗p < 0.05; ∗∗∗p < 0.001. For all both boxplots, each dot represents individual replicate, the center values show the medians, the boxes indicate the first and third quartiles and the bars the 10^th^ and 90^th^ percentiles. See also Figure S6.

Finally, we sought to determine the functional significance of poly(A) tail processing on cell proliferation and differentiation. To this end, we artificially induced in undifferentiated cells the poly(A) tail dynamic observed during differentiation by activating the mTOR pathway with MHY1485. After 48 h of MHY1485 treatment, we observed enlarged autophagosomes characteristic of the inhibitory effect of MHY1485 on autophagy (Figure S6A). However, the treated undifferentiated cells showed no other morphological changes characteristic of neuronal differentiation when compared to control cells exposed to DMSO (Figure S6A). Furthermore, the neuronal differentiation marker *Map2* was not differentially expressed in response to MHY1485 (Figure S6B). These results indicate that the dynamic regulation of TOP mRNA poly(A) tails alone is not sufficient to trigger cell differentiation. In addition, premature poly(A) tail processing of TOP transcripts did not prevent cell differentiation in the presence of retinoic acid (Figure 6A). In fact, the induction of the differentiation marker *Map2* is even higher for cells differentiated in the presence of MHY1485 than for control cells (Figure 6B), suggesting a premature entry into differentiation. Consistently, MHY1485 treatment significantly reduced the proliferation of undifferentiated cells and, to a lower extent, the proliferation of D2 differentiating cells (Figure 6C). Together, our results support the notion that the timely processing of TOP mRNA poly(A) tails indirectly regulates neuronal differentiation by maintaining the pool of progenitor cells, which ultimately define the number of differentiated neurons.

## Discussion

Stem cell differentiation is associated with global changes in protein synthesis.^1^ TOP transcripts expression supports the translational program of differentiating cells by providing the resources to create functional ribosomes. Mutations to genes encoding TOP transcripts lead to adverse phenotypes known as ribosomopathies which impact various stem cell compartments, including neuronal progenitors.^7^ In addition, patients with ribosomopathies show susceptibility to cancer, supporting the association between translational dysregulation and differentiation defects.^8^ Here, we showed that a specific poly(A) tail-length processing uncovers the stability and translational activation of TOP mRNAs during neuronal differentiation.

Poly(A) tail length processing is tightly linked to the stability and translational activation of transcripts.^20^ However, under steady-state conditions, transcripts with vastly different half-lives and translational efficiencies have similar poly(A) tail lengths.^25^^,^^35^ An association between poly(A) tail length and translational activity has only been observed in systems where transcription is paused, and mRNAs are particularly stable. This is the case, for example, of early embryogenesis where translational activation correlates positively with poly(A) tail length^26^ or spermiogenesis where transcripts become translationally active only when their poly(A) tails shorten from ∼150-nts to ∼60-nts.^27^^,^^28^ In our study, we measured poly(A) tail-length dynamics in a model system of early neuronal differentiation. The analysis of successive differentiation stages for mRNA synthesis, decay and translational activity at the transcriptome-wide level allowed us to uncover how the specific poly(A) tail processing of TOP mRNAs is linked to their stability and translational activation during neurogenesis. Our system resembles post-transcriptional regulation in spermatids where transcripts become translationally active as they accumulate with ∼60-nt long tails.

TOP mRNA metabolism has been extensively studied in contexts other than mammalian cell differentiation.^35^^,^^44^^,^^45^^,^^46^ In the late 1980s, a study in *Xenopus laevis* already proposed mRNA stability as a layer of RP regulation.^47^ Our cell system allowed us to investigate the role of TOP mRNA stability during the process of neuronal differentiation. As the cells differentiate, we observed a global acceleration of decay affecting both TOP and non-TOP transcripts. However, TOP mRNAs are highly stable before and throughout differentiation compared to the rest of the transcriptome. Consequently, the downregulation of TOP mRNAs is relatively subtle compared to other downregulated transcripts with a faster turn-over. Thus, given that the average half-life of the TOP transcripts is ∼20 h for undifferentiated cells, a significant fraction of TOP transcripts synthesized before the onset of differentiation is still present as cells differentiate.

Poly(A) tail shortening is a mandatory step for mRNA decay.^24^ However, the importance of poly(A) tail processing in defining TOP mRNA stability has only been recognized recently.^16^^,^^17^^,^^18^^,^^19^ mRNA degradation relies on the sequential activity of 3′ deadenylases. Newly transcribed mRNAs have poly(A) tails ∼250-nt long coated by multiple PABPs which are progressively peeled off as the PAN2/3 deadenylase complex shortens the poly(A) tail.^48^ Once tails reach ∼110-nts, PAN2/3 are less efficient and the deadenylation is continued by the CNOT complex to ultimately shorten the tails down to ∼30-nts, after which the mRNA is rapidly degraded.^48^^,^^49^ In this study, we found that the accumulation of transcripts with tails <30-nt long correlates with decay acceleration. However, TOP mRNAs deviate from this general pattern. Although TOP mRNA decay rates are comparable to other downregulated transcripts during differentiation, they accumulate with <30-nt long tails in a higher proportion. Similar observations have been reported in *C. elegans*, where abundant mRNAs remain stable in a short-poly(A)-tailed state.^50^

Several studies have shown that TOP mRNA stability relies on LARP1.^13^^,^^15^^,^^16^^,^^17^^,^^18^^,^^19^ Our investigations further support previous observations, highlighting the association between LARP1’s role in poly(A) tail processing and transcript stability. We found that in progenitor cells, LARP1 prevents the whole transcriptome from deadenylation and provides additional protection to TOP mRNA poly(A) tails. How LARP1 shapes TOP mRNA poly(A) tail is an active area of research. An initial study proposed a role for LARP1 in promoting 3′ polyadenylation of TOP mRNAs.^17^ Recently, LARP1 was shown to physically block the interaction of TOP transcript with the deadenylase complex CNOT.^16^

The resistance of TOP mRNA tails to deadenylation is believed to favor further post-transcriptional regulation upon them.^16^^,^^17^ RP mRNAs and other abundant transcripts have been shown to preferentially accumulate with poly(A) tails ∼60-nt long.^50^ In this study, we show that the TOP mRNAs poly(A) tail distribution is not a fixed property but rather changes dynamically as stem cells differentiate into neurons. During this process, TOP mRNA accumulates with poly(A) tails ∼60-nt long while simultaneously becoming more translationally active. Consistently, the activation of mTOR, known to favor TOP mRNA translation, is sufficient to partially recapitulate the accumulation of TOP mRNA with ∼60-nt long tails. Here we propose that TOP mRNAs have increased deadenylation rates of poly(A) tails ∼120-nt long upon activation of the mTOR pathway and reduced deadenylation rates of tails ∼60-nt long mediated by LARP1. Both mechanisms likely contribute simultaneously to the accumulation of transcripts with ∼60-nt long tails.

The accumulation of TOP mRNAs with ∼60-nt long poly(A) tails occurs early on during differentiation suggesting that a significant pool of TOP transcripts inherited from progenitor cells are actively modified post-transcriptionally to support differentiation. This observation is reminiscent of TOP mRNA tail processing in other cellular contexts where the translational machinery needs to drive rapid cell adaptation in response to stress conditions.^17^^,^^51^ Here, we propose that the specific poly(A) tail dynamic of TOP mRNAs support their stability through differentiation and enhance their translational upon mTOR activation; we hypothesize that this process is essential to generate the ribosomes required to complete the synthesis of the neuronal proteome, while the production of TOP mRNAs is repressed transcriptionally. Indeed, post-transcriptional mechanisms are known safeguards to ensure the continuity of essential cellular processes while a new transcription program is established.^52^^,^^53^

The uncoupling between high protein synthesis and activation of ribosome production was observed in other systems of stem cell differentiation.^54^ Controlling these metabolic activities is critical to ensure the balance between self-renewal and differentiation.^2^^,^^55^^,^^56^ Here we show that the premature induction of TOP mRNA poly(A) tail processing in undifferentiated cells upon mTOR activation did not prevent them from differentiating in the presence of retinoic acid. However, mTOR activation reduces progenitor cell proliferation. Similar conclusions have been recently reached by Gui and collaborators using a model of germ cell differentiation in Drosophila.^57^ Our results support a model where TOP mRNA poly(A) tail processing contributes to control the sensitive balance between self-renewal and differentiation.

Several therapies have been designed to restore normal ribosome biogenesis in the context of congenital ribosomopathies or cancers.^58^ Most of them are designed to trigger TOP mRNA cap-dependent translation or rRNA transcription. We envision that further studies dissecting the pathways influencing TOP RNA poly(A) tail processing will create exciting opportunities to treat conditions associated with ribosome biogenesis dysregulation.

### Limitations of the study

In this study, we used P19 cells as a model for neuronal differentiation. Our observations, however, may not extend to every *in vitro* or *in vivo* system of neurogenesis. Other neuronal cell types may behave different in terms of LARP1 regulation and the timing of mTOR activation. At the molecular level, the high stability of TOP transcripts limits the methods available to study their decay rates. Treatment with actinomycin D not only induces cell death due to sustained transcriptional silencing but also can trigger a stress response specifically affecting RPs. Therefore, in our study, we only assessed transcript stability using 4sU metabolic labeling. Finally, the mTOR complex can influence transcriptional and post-transcriptional pathways. Although we observed an accumulation of TOP mRNAs with ∼60-nt long tails and reduce cell proliferation upon mTOR activation, we cannot rule out other regulatory mechanisms driving the limited replication capacity of progenitor cells.

## STAR★Methods

### Key resources table

**Table:** 

REAGENT or RESOURCE	SOURCE	IDENTIFIER
**Antibodies**		

Rabbit mAb Phospho-S6 Ribosomal Protein (Ser235/236)	Cell Signaling Technology	Cat# 4858; RRID:AB_916156
Rabbit mAb S6 Ribosomal Protein	Cell Signaling Technology	Cat# 2217; RRID:AB_331355
Rabbit pAb LARP1	Thermo Fisher Scientific	Cat# A302-087A; RRID:AB_1604274
Goat Anti-Mouse IgG Antibody, Fc, HRP conjugate	Millipore SigmaAldrich	Cat# AP127P; RRID:AB_92472
Goat Anti-Rabbit IgG Antibody, Fc, HRP conjugate	Millipore SigmaAldrich	Cat# AP156P; RRID:AB_91699

**Chemicals, peptides, and recombinant proteins**		

Retinoic acid	Sigma	Cat# R2625 CAS: 302-79-4
TaKaRa NDiff® 227 Medium	Takara	Cat# Y40002
O-Propargyl-puromycin	Cayman Chemical	Cat# 13884 CAS: 1416561-90-4
Cycloheximide	Sigma-Aldrich	Cat# C7698 CAS: 66-81-9
Dimethyl sulfoxide	Sigma-Aldrich	Cat# D2650 CAS: 67-68-5
MHY1485	Selleckchem	Cat# S7811 CAS: 326914-06-1

**Critical commercial assays**		

CellTiter-Glo® Luminescent Cell Viability Assay	Promega	Cat# G7571
Click-iT™ EdU Alexa Fluor™ 488 Flow Cytometry Assay Kit (50 assays)	Thermo Fisher Scientific	Cat# C10425
Click-iT™ Plus OPP Alexa Fluor™ 647 Protein Synthesis Assay Kit	Thermo Fisher Scientific	Cat# C10458
Pierce™ BCA Protein Assay Kit	Thermo Fisher Scientific	Cat# 23225
SLAMseq Kinetics Kit - Catabolic Kinetics Module	Lexogen	Cat# 062.24
SLAMseq Kinetics Kit – Anabolic Kinetics Module	Lexogen	Cat# 061.24
QuantSeq 3′ mRNA-Seq Library Prep Kit FWD for Illumina	Lexogen	Cat# 015.24
QIAquick PCR purification kit	Qiagen	Cat# 28104
RNA Clean & Concentrator-5	Zymo research	Cat# R1013
Direct RNA Sequencing Kit	Oxford Nanopore Technologies	Cat# SQK-RNA002

**Deposited data**		

Raw and analyzed data	This paper	GEO: GSE214875, GSE231980
WB RAW images	This paper	Upon request
Microscopy pictures	This paper	Upon request
GENCODE vM23	Gencode	https://www.gencodegenes.org/mouse/release_M23.html
GENCODE vM17	Gencode	https://www.gencodegenes.org/mouse/release_M17.html

**Experimental models: Cell lines**		

Mouse embryonal carcinoma P19 cells	ATCC	Cat# CRL-1825, RRID: CVCL_2153

**Oligonucleotides**		

ON-TARGETplus Non-targeting Control Pool	Dharmacon	Cat# D-001810-10-20
ON-TARGETplus Mouse Larp1 siRNA	Dharmacon	Cat# J-063706-09-0010
Map2_fw: CGAAGGATAAAGTCACTGATGG Map2_rev: GCTCTGCGAATTGGTTCTGACC	This paper	N/A
Act_fw: GATGTATGAAGGCTTTGGTC ß-Act_rev: TGTGCACTTTTATTGGTCTC	This paper	N/A
Barcoded oligonucleotides for RNA poly(A) standards, see Table S9	This paper	N/A
Primers for Direct RNA multiplexing, see Table S10	This paper	N/A

**Recombinant DNA**		

pSpCas9(BB)-2A-GFP (PX458) plasmid	Addgene	RRID: Addgene_48138

**Software and algorithms**		

GraphPad, Prism Software	Dotmatics	N/A
NIS-Elements Imaging Software	Nikon	N/A
ABI PRISM 7900 Sequence Detection System and Analysis Software	Applied Biosystems	N/A
MinION/GridION analysis software	Oxford Nanopore Technologies	N/A
FlowJo Solftware	bdbiosciences	N/A
Ingenuity Pathway Analysis (IPA) software	Qiagen	N/A
R-studio software Version 2022.12.0 + 353	Posit Software, PBC	N/A
BiomaRt	Durinck et al.,^65^	https://bioconductor.org/packages/release/bioc/html/biomaRt.html
Multiple EM for Motif Elicitation (MEME)	Bailey and Elkan,^66^	https://meme-suite.org/
GO annotation of Biological Processes	Ashburner et al.,^67^ Gene Ontology Consortium,^68^	http://geneontology.org/
TopGO R package	https://doi.org/10.18129/B9.bioc.topGO	https://bioconductor.org/packages/release/bioc/html/topGO.html
bcl2fastq v2.20 software	Illumina	https://support.illumina.com/downloads/bcl2fastq-conversion-software-v2-20.html
BBTools v38.96	Brushnell et al.,^69^	https://sourceforge.net/projects/bbmap/
Xtail v1.1.5	Xiao et al.,^70^	https://github.com/xryanglab/xtail/releases
Illumina software bcl2fastq v2.20	Illumina	https://support.illumina.com/downloads/bcl2fastq-conversion-software-v2-20.html
SLAMdunk analysis pipeline	Neumann et al., 2019	https://t-neumann.github.io/slamdunk/
extract-transcript-regions	N/A	https://github.com/stephenfloor/extract-transcript-regions
biomaRt	Durinck et al.,^65^	https://bioconductor.org/packages/release/bioc/html/biomaRt.html
Guppy	Oxford Nanopore Technologies	https://nanoporetech.com/how-it-works/basecalling
Minimap2 software v2.20-r1061	Li,^71^	https://github.com/lh3/minimap2/releases
Nanopolish	Loman et al.,^43^	https://github.com/jts/nanopolish
SAMtools	Danecek et al.,^72^	https://github.com/samtools/samtools
Deeplexicon	Smith et al.,^64^	https://github.com/Psy-Fer/deeplexicon
DESeq2	Love et al.,^74^	https://bioconductor.org/packages/release/bioc/html/DESeq2.html
Cumulative distribution function (CDF) of the hypergeometric distribution	Graeber Lab	https://systems.crump.ucla.edu/hypergeometric/
ΔΔCt method	Rao et al.,^62^	

**Other**		

Neuronal differentiation	Morii et al.,^61^	
MIQE standards	Bustin et al.,^63^	
TOP-motif mRNAs curated from	Cockman et al.,^73^	

### Resource availability

#### Lead contact

Further information and requests for resources and reagents should be directed to and will be fulfilled by the lead contact, Marcos Morgan (marcos.morgan@nih.gov).

#### Materials availability

This study did not generate new unique reagents.

### Experimental model and study participant details

#### Cell culture

Male mouse embryonal (E7) carcinoma P19 cells^60^ (ATCC, CRL-1825) were maintained in Dulbecco’s modified Eagle medium with 4,500 mg/L of glucose supplemented with 100 units/mL of penicillin/streptomycin (Life Technologies) and 10% fetal bovine serum (FBS, Gemini Bio-Products). For culture, cells were incubated at 37°C and 5% CO_2_ atmosphere. All experiments have been conducted with original ATCC cells not exceeding eight passages. To induce neuronal differentiation, 3 x 10^5^ cells were seeded in poly-L-lysine-coated 6-well plates in TaKaRa NDiff® 227 Medium (Takara) with retinoic acid (500 nM; Sigma) as previously described.^61^ Media was changed daily up to 6 days after induction. Morphological changes were visualized using the brightfield channel of the Nikon Eclipse TS2-fl microscope (Nikon Instruments Inc.).

### Method details

#### Cell treatment and transfection

The mTOR pathway activator MHY1485 (Selleckchem) was prepared as a 10 mM stock solution in DMSO and stored at -80°C. The day after seeding, undifferentiated P19 cells were treated with 10 μM final concentration of MHY1485 for 24 or 48 hours with a fresh media change every 24 hours. Cells exposed to the same amount of DMSO were used as control. To study the impact of mTOR activation on differentiation, undifferentiated P19 cells treated for 48 hours with 10 μM MHY1485 or DMSO were harvested and seeded at 200,000 cells per well in 6-well plates. The cells were then grown in either maintenance or differentiating conditions for 48 hours in the presence of DMSO or 10 μM MHY1485 to ensure the continuity of the initial exposure. To knock down *Larp1*, undifferentiated P19 cells seeded in 6-well plates were transfected with 50 nmoles of *Larp1* siRNA (Dharmacon, J-063706-09-0010) using Lipofectamine 3000 (Invitrogen) according to the manufacturer's instructions. A pool of scrambled siRNAs (Dharmacon, D-001810-10-20) with no significant homology to the human, mouse, or rat genome was used as negative control. After transfection, cells were incubated at 37°C for 24 hours. To study the long-term effect at 48 hours, an additional transfection using the same conditions was performed 24 hours after the initial knock-down. Morphological changes were visualized using the brightfield channel of the Nikon Eclipse TS2-fl microscope (Nikon Instruments Inc.).

#### Cell viability

The CellTiter-Glo luminescent cell viability assay was performed according to themanufacturer’s instructions (Promega). Undifferentiated (D0) and differentiated (D6) P19 cells were treated with a two-fold serial dilution of 4sU ranging from 0 to 4000 μM for a total of 27 hours of exposure prior to collection. Media was changed with fresh 4sU at 15 hours and 3 hours before harvesting the cells to ensure the continuity of the exposure. Cells were then incubated for 10 minutes with CellTiter-Glo reagent and placed on an orbital shaker. The content of each well was transferred to an opaque walled plate and the luminescent signal was measured using a 96-well plate reader (GloMax-96 microplate luminometer, Promega). The 4sU concentrations associated with 90% cell viability were interpolated from the sigmoidal fit of the standard dilution. To control for the linearity of the assay, a serial dilution of cell seeding was used. Media without cells was used to measure and subtract the background signal for downstream analysis.

#### Cell proliferation

Proliferation was measured by EdU incorporation using the Click-iT EdU Alexa Fluor 488 Cell Proliferation Assay Kit according to the manufacturer’s protocol (Thermo Fisher Scientific). Briefly, P19 cells were incubated in medium supplemented with 10 μM EdU for 2 hours. Cells were harvested, washed with PBS-1% BSA, fixed with 100 μL of Click-iT fixative solution for 15 minutes and then permeabilized with 100 μL of Click-iT saponin-based permeabilization buffer for 15 minutes at room temperature. Cells were mixed with a Click-iT reaction cocktail containing the Alexa Fluor 488 for 30 minutes to allow detection of EdU fluorescence labeling. After three washing steps, cells were resuspended in 150 μL saponin-based permeabilization buffer and analyzed by flow cytometry. Forward and side scatter gating was used to select a homogenous population of cells. EdU fluorescence was captured in the FITC channel. Background signal was assessed by cells not exposed to EdU. The percentage of cells showing a positive signal for EdU was quantified based on two thousand events per sample in two to three biological replicates per condition for 2 independent experiments.

#### Global protein synthesis

P19 cells were incubated in medium supplemented with 20 μM O-propargyl-puromycin (OPP, Cayman Chemical). After 30 minutes, cells were harvested, washed with PBS, fixed with 4% PFA for 15 minutes and then permeabilized with 0.5% Triton X-100 for 15 minutes at room temperature. OPP fluorescence labeling was induced using the Click-iT Plus OPP Alexa Fluor 647 Protein Synthesis Assay Kit according to the manufacturer’s protocol (Thermo Fisher Scientific). After three PBS washes, cells were resuspended in PBS and analyzed by flow cytometry. Forward and side scatter gating was used to select a homogenous population of cells. OPP fluorescence was captured in the Cy5 channel. Two thousand events per sample were considered for analysis in three biological replicates per condition. Signal specificity was assessed by cotreatment with 100 μM cycloheximide (CHX, Sigma) or in the absence of OPP (Figure S7A).

#### Western blotting

Cells were washed with PBS and protein lysates were prepared using RIPA Lysis and Extraction Buffer (Thermo Fisher Scientific) with the addition of Protease and Phosphatase Inhibitor Cocktail (Thermo Fisher Scientific) to prevent proteolysis and maintain protein phosphorylation. Protein concentrations were determined with the BCA Protein Assay Kit according to the manufacturer’s protocol (Pierce) using bovine serum albumin (BSA) for standards. 30 μg of protein extract per lane were separated on 4–20% polyacrylamide gels (Biorad) and transferred to nitrocellulose membranes (0.2 μm pore size) for 13 minutes using a dry Gel Transfer Device (iBlot™). Membranes were blocked for 1 hour in Tris-buffered saline (TBS)-0.5% Tween-20 containing either 5% non-fat milk or 5% bovine serum albumin when detecting phosphorylated proteins. Membranes were incubated with anti-Phospho^Ser235/236^-ribosomal protein S6 antibody (Cell signaling, 4858, 1/2000 dilution), anti-ribosomal protein S6 antibody (Cell signaling, 2217, 1/2000 dilution) or anti-LARP1 antibody (Thermo Fisher Scientific /Life tech, A302-087A, 1/2000 dilution) at 4°C overnight. Membranes were washed three times in PBS for ten minutes, prior to the incubation with horse radish peroxidase-conjugated secondary antibodies (Millipore SigmaAldrich) and development with ECL (Thermo Fisher Scientific) using the AMERSHAM ImageQuant800 imaging system (Cytiva).

#### RNA extraction

Cells were washed with PBS and collected in 1 mL of TRIzol. Five minutes later, 200 μL of chloroform-isoamyl alcohol were added. Then, the mixture was vigorously shaken, incubated for 2 minutes at room temperature, and centrifuged at 12,000 g for 15 minutes at 4°C. The aqueous fraction was transferred to a new tube, and RNA was recovered by adding 400 μL isopropanol. After 15 minutes, the samples were centrifuged at 12,000 g for 10 minutes at 4°C. The RNA pellet was washed with 75% ethanol and then centrifuged at 7,500 g for 5 minutes at 4°C. After ethanol removal, total RNA was resuspended in nuclease-free water and quantified using a Nanodrop 2000 spectrophotometer (Thermo Fisher Scientific). RNA integrity was confirmed on the bioanalyzer system Agilent 2100 (Agilent Technologies) prior to further analysis.

#### qPCR

Complementary DNA (cDNA) was synthesized from 1 μg of DNase-treated (Qiagen) total RNA using random hexamer primers and the SuperScript™ II Reverse Transcriptase reaction mix prepared according to the manufacturer’s protocol (Invitrogen). qPCR was performed using SYBR Green mix (Applied Biosystems). Briefly, 2.5 μL of 10 times diluted cDNA were added to 17.5 μL of the enzymatic reaction per well according to the manufacturer’s protocol. The cycling conditions were as follows: 95°C for 30 seconds; 40 cycles of 95°C for 15 seconds, and 60°C for 30 seconds. The amplification was measured and analyzed using the ABI PRISM 7900 Sequence Detection System and Analysis Software (Applied Biosystems). The primer sequences used are: Map2_fw: CGAAGGATAAAGTCACTGATGG, Map2_rev: GCTCTGCGAATTGGTTCTGACC, ß-Act_fw: GATGTATGAAGGCTTTGGTC, β-Act_rev: TGTGCACTTTTATTGGTCTC. For each reaction, standard curves were generated, and amplification efficiencies were calculated based on the slope of the regression between the standard concentrations and their Ct value. Water was used as a non-template control and a dissociation curve (60°C–95°C) was used to control for specific amplification. Quantification of expression was calculated using the ΔΔCt method.^62^ This study was performed in compliance with MIQE standards.^63^

#### RNA synthesis and decay rates

To measure RNA synthesis and decay rates, the anabolic and catabolic modules of the SLAM-seq kinetics kits were used according to the manufacturer’s instructions (Lexogen). Briefly, the transcriptomes of undifferentiated (D0) and differentiated (D6) P19 cells were metabolically labeled with a solution of 20 μM and 500 μM 4sU, respectively. These concentrations were shown to maintain the metabolic status of 90% of the cells after 27 hours of exposure considering that a maximum of 15 hours incubation would be used for the experiment (Figure S7B). For RNA synthesis, nascent RNAs were labeled and collected at 0, 3, 6, and 9 hours after 4sU exposure. For RNA decay, the transcriptome was saturated with 4sU labeling for a total of 15 hours, with the addition of a fresh solution of 4sU 3 hours before the end of the exposure. To terminate the 4sU labeling, cells were washed with PBS, and maintained on media with a 10 mM UTP concentration. To track the decay of labeled RNA, cells were collected at 0, 3, 6, and 9 hours after UTP addition. Total RNA was extracted using standard TRizol procedure protected from direct light to prevent 4sU from crosslinking. IAA treatment was performed on up to 5 μg of total RNA on three replicates per timing. RNA concentration and integrity were measured prior to library preparation. Libraries were built and multiplexed using the QuantSeq 3′ mRNA-Seq Library Prep Kit for Illumina (Lexogen) following the manufacturer guidelines. Libraries were pooled in equimolar amounts and sequenced on a NovaSeq 6000 sequencer (Illumina) using an S1 Flow Cell, on a 101bp single-end mode. Read number obtained per replicate range from 26,801,031 to 68,718,129 for an average of 40,349,093 reads prior to analysis. The global correlation between changes in synthesis or decay rates during differentiation and differential RNA accumulation was used as a validation of SLAM-seq results; downregulated transcripts showed the strongest slow-down in synthesis parallel to the fastest decay during differentiation, while upregulated transcripts showed opposite dynamics (Figures S7C and S7D)

#### Synthesis of poly(A) standards

RNA poly(A) standards were synthesized using barcoded oligonucleotides with encoded tail lengths or barcoded oligonucleotides without encoded tail lengths (Table S9). For standards generated using oligonucleotides without encoded tails, tails were added by *in vitro* polyadenylation as described below. Oligonucleotides were used to amplify the same fragment of the pSpCas9(BB)-2A-GFP (PX458) plasmid encoding the GFP open reading frame. In every case, the same forward plasmid was used, which contained the T7 RNA polymerase promoter sequence. PCR reactions of 100 μL were prepared for each amplification using 1 μL Phusion polymerase (NEB) and final concentrations of 1X Phusion polymerase buffer (NEB), 200 μM dNTPs (Thermo Fisher Scientific), 1 μM reverse and forward primers (Table S9), and 1 pg/μL of template vector. The amplification reactions consisted of a step of initial denaturation at 95°C for 3 minutes, followed by 34 cycles of 30 seconds at 95°C, 30 seconds at 58°C, and 2 minutes at 72°C and a final extension step at 72°C for 30 minutes. The amplicon was then purified with the QIAquick PCR purification kit according to manufacturer’s instructions (Qiagen). The RNA standards were then synthesized using the purified amplicons as templates for the T7 RNA polymerase (NEB). The RNA synthesis reaction consisted of 1X of T7 RNA polymerase mix, 1X Reaction buffer, ATP, CTP, GTP, and UTP 10 mM each (Thermo Fisher Scientific), and 1 μg of the template in a final volume of 20 μL. The reaction was then incubated for 2 hours at 37°C. The RNA was purified using the RNA Clean & Concentrator Zymo kit according to manufacturer’s instructions (Zymo research). The purified standards were eluted in 30 μL of Nuclease-free water.

#### *In vitro* polyadenylation assay

To add poly(A) tails to RNA standards without an oligonucleotide-encoded tail, the *E. coli* poly(A) polymerase (NEB) was used. The reaction mix for each standard was prepared by adding 6 μL of 10X *E. Coli* Poly(A) Polymerase Reaction Buffer, 6 μL of ATP 10 mM, 1.5 μL of *E. coli* poly(A) polymerase, and 16.5 μL of nuclease-free water. The mix was then split into three aliquots; 10 μL of the RNA standard 1 μg/μl were then added to each aliquot. The standards were incubated for ∼15 or 30 minutes at 37°C to obtain different poly(A) tail lengths. The aliquots for each standard were then pooled and purified using the RNA Clean & Concentrator Zymo kit following the manufacturer’s instructions.

#### Poly(A) tail purification

For library preparation, mRNA from P19 cells before (D0) and during differentiation (D2, D4, and D6) were purified from total RNA using Dynabeads™ Oligo(dT)25 (Thermo Fisher Scientific). Briefly, 20 μL of beads per sample were washed, resuspended in 40 μL of binding buffer (20 mM Tris-HCl pH 7.5, 1.0 M LiCl, and 2 mM EDTA) and mixed with an equivalent volume of water containing 3.5 μg of denatured RNA. After 5 minutes of incubation at room temperature, beads were collected on a magnetic rack and the supernatant was removed. The beads were washed twice in a wash buffer (10 mM Tris-HCl, pH 7.5, 0.15 M LiCl, and 1 mM EDTA) and the mRNA was eluted after a 2-minute incubation at 80°C in 10 μL of Tris-HCl, pH 7.5 for library preparation.

#### Library multiplexing

Direct RNA multiplexing was designed as previously described.^64^ Briefly, four distinct barcodes (BC 1–4) were included in the sequence of the oligonucleotides A and B used for the initial ligation step of the Direct RNA Sequencing protocol from ONT (Direct RNA Sequencing Kit- SQK-RNA002). Oligonucleotide sequences are listed in Table S10. To create a primer duplex, the corresponding oligonucleotides A and B (final concentration of 1.4 μM each) were incubated together at 94°C for 5 minutes in a total volume of 75 μL of annealing buffer (0.01 M Tris-HCl pH 7.5, 0.05 M NaCl). To favor annealing, the reaction was gradually cooled down to room temperature (-0.1°C/second).

#### Direct RNA sequencing

Direct RNA sequencing libraries were prepared according to ONT direct RNA sequencing protocol (SQK-RNA002) with modifications to incorporate the barcoded oligonucleotides. For each time point of cell differentiation (D0, 2, 4, and 6), independent libraries were prepared by multiplexing three biological replicates using the barcoded oligonucleotides BC1, 2, and 3. For DMSO and MHY1485 at 24 or 48 hours, two independent libraries were prepared for each time point by multiplexing two biological replicates per condition using the barcoded oligonucleotides BC1, 2, 3 and 4. For siCtl and siLarp1 at 24 h, two independent libraries per condition were prepared by multiplexing three biological replicates using the barcoded oligonucleotides BC1, 2, and 3. For siCtl and siLarp1 at 48 h, one library was prepared multiplexing two biological replicates per condition using the barcoded oligonucleotides BC1, 2, 3 and 4. To capture the full poly(A) length, the 3′ ends of the transcripts were splint ligated to the barcoded oligonucleotides. For this, 3 μL of 5X ligation buffer (6% PEG in NEB T4 DNA ligation buffer), 1.5 μL of T4 DNA ligase (NEB), 1 μL of 0.1 ng/μL RNA standard mix, and 1.5 μL of annealed oligonucleotides (100 nM) were mixed with 9 μL of poly(A) selected RNA for a final reaction volume of 30 μL. The ligation reaction was incubated at room temperature for 10 minutes. To maximize the sequencing efficiency, RNA-cDNA hybrids were generated by reverse transcription according to manufacturer’s instructions (Thermo Fisher Scientific). Briefly, 30 μL of splint ligated RNA was mixed with 9 μL of nuclease-free water, 2 μL of dNTPs (10 mM), 8 μL of 5X first strand buffer, 4 μL of 100 mM DTT, and 2 μL of SuperScript II reverse transcriptase (Thermo Fisher Scientific). The reaction was then incubated at 50°C for 50 minutes followed by 10 minutes at 70°C and cooled at 4°C. The RNA/cDNA hybrids were purified with AMPure beads (Beckman Coulter) and washed with ethanol 70%. For multiplexing, three replicates per timing with distinct barcodes were eluted together after 5 minutes at room temperature in 20 μL of nuclease-free water. Sequencing adapters supplied in the kit were attached to the ends of the multiplexed RNA/cDNA hybrids. Briefly, 8 μL of 5X ligation buffer (6% PEG in NEB T4 DNA ligation buffer), 6 μL of RMX-RNA adapter, 3 μL of nuclease-free water, and 3 μL of T4 DNA Ligase (NEB) were added to 20 μL of purified RNA/cDNA hybrid for a final reaction volume of 40 μL. The ligation reaction was incubated for 15 minutes at room temperature. The ligation products were purified with AMPure beads (Beckman Coulter), washed twice with 150 μL of wash buffer (WSB from SQK-RNA002), and eluted in 21 μL of elution buffer (EB from SQK-RNA002) for 5 minutes at room temperature. Libraries were loaded in ONT flow cells (R9) using Flow Cell Priming Kit (EXP-FLP002) according to the manufacturer's instructions. Briefly, 37.5 μL of RNA running buffer (from SQK-RNA002) and 17.5 μL of nuclease-free water were added to 21 μL of adaptor-ligated libraries. The flow cell was primed by adding 800 μL of the FLT/FB mix (30 μL FLT for 1 mL of FB, from EXP-FLP002) into the priming spot, followed by an additional 200 μL after 5 minutes incubation. Finally, 75 μL of the library was loaded through the spot-on port and sequenced on MinION or GridION instrument (ONT). For sequencing poly(A) tails during differentiation, the number of mapped reads obtained per replicate ranged from 163,168 to 829,567 for an average of 501,425 reads prior to analysis. For sequencing poly(A) tails from undifferentiated cells exposed to DMSO or MHY1485, or transfected with siCtl or siLarp1, the number of mapped reads obtained per replicate ranged from 146,734 to 557,313 for an average of 282,885 reads prior to analysis.

### Quantification and statistical analysis

#### Multiple expectation maximizations for motif Elicitation (MEME) analysis

The UTR sequences of transcripts showing statistical accumulation with <30- or ∼60-nt long poly(A) tails during differentiation were retrieved from the annotation file mmusculus_gene_ensembl using the getSequence function from the biomaRt package.^65^ To format the sequences for RNA motif discovery, the Ts from DNA sequences were substituted by Us. The first 100 nucleotides at the extremities of each UTR were converted to FASTA and exported as input for the motif discovery tool MEME available at https://meme-suite.org/.^66^ The motif query was set for the given strand with a minimum width of 5 to 20 nucleotides. Results were limited to the three most enriched motifs. E-values were calculated for the log likelihood ratio (LLR) of the occurrences of the motif corrected for multiple testing.

#### Gene ontology analysis

The ontology enrichment analyses were done using the TopGO R package (https://doi.org/10.18129/B9.bioc.topGO). Briefly, the gene universe, accounting only for genes captured on our sequencing analyses, and genes of interest were selected from each experiment. The corresponding TopGOdata object was built using “annFUN.org” for the Biological Processes gene ontology annotation.^67^^,^^68^ To run the enrichment analysis, the function runTest was used with a classic algorithm considering each gene ontology category independently, and a Fisher’s exact test for statistical enrichment. Finally, the GenTable function was used to generate a table with the 10 most significant gene ontology terms and their corresponding p-values (Figure 1E). The full list of significant terms is detailed in Tables S2 and S7. Ingenuity Pathway Analysis (IPA) software (Qiagen) was used for the functional core analysis of transcripts with differential accumulation (DESeq2 p-value <0.05) or differential translational engagement (Xtail log_2_ fold change > 0.5). The predicted upstream regulators were filtered to only consider the molecule type “transcription regulator” when analyzing differential accumulation. For the differential translational engagement analysis, the upstream regulators were filtered to consider “translation regulator”. The 5 most significantly enriched canonical pathways or upstream regulators and their corresponding activation z-score were used to generate Figures S1C and S3C. Full reports are detailed in Tables S4 and S8.

#### Ribo-seq analysis

The reads base calling was done using the bcl2fastq v2.20 software (Illumina). Optical duplicates were removed with the clumpify tool of BBTools v38.96^69^. The BBDuck tool, also part of the BBTools suite, was used for adapter trimming, rRNA removal and read filtering. The read filtering range was 20 to 50 nucleotides and 26 to 34 nucleotides for the RNA-seq and RFP libraries, respectively. Filtered reads were mapped to the GENCODE vM23 transcripts (mm10 assembly) with the BBMap tool, also part of the BBTools suite, using the slow mode. A minimal pre-filtering was performed to only consider the contigs showing a minimum of 30 reads across replicates and conditions for further analysis. The differential translational engagement was determined with Xtail v1.1.5^70^ using read counts of RNA-seq and RFP libraries from D0 and D6 samples as input.

#### SLAM sequencing analysis

Base calling was done with the software bcl2fastq v2.20 (Illumina). To quantify T to C conversions after 4sU treatment, the SLAMdunk analysis pipeline was used as described.^42^ Briefly, the input 3’ UTR bed file was prepared using the extract_transcript_regions tool (https://github.com/stephenfloor/extract-transcript-regions) with the mm10 assembly and GENCODE vM17 GTF (GENCODE) as input. The single nucleotide polymorphisms were called on the aligned reads using the snp tool, followed by the quantification of T to C conversions with the count tool. For the anabolic and catabolic assay, read conversion was normalized to the maximum conversion rate expected for the same transcript after 13 hours of 4sU labeling (time 0 of the catabolic time course). The half-lives of synthesis and decay were estimated by exponential fit using the R function nonlinear least squares (nls). Only R squared values > 0.6 were considered for interpretation. Half-life values <0 or >5000 minutes were considered as outliers and ignored for further analysis.

#### Direct RNA sequencing analysis and poly(A) tail length determination

Reads base calling was done with Guppy (ONT). The reads were then aligned to the mm10 assembly annotated with GENCODE vM17 genes using Minimap2 software v2.20-r1061, with the -x parameter set to map-ont.^71^ The length of the poly(A) tail for each read was determined using Nanopolish.^43^ To run the Nanopolish poly(A) pipeline, reads were indexed with Nanopolish and mapped reads were sorted and indexed with SAMtools.^72^ Only reads with poly(A) tail PASS quality score were considered for further analysis. Reads were demultiplexed using Deeplexicon.^64^ 90% of the reads were above a confidence interval of 0.75. The reads were also aligned to the spike-ins and high-quality mapping spikes-ins were retained to ensure correct separation of the barcodes. The spike-ins were grouped by barcodes and their cumulative frequency of poly(A) length was plotted (Figure S7E). A minimal pre-filtering was performed to ignore transcripts encoded by the mitochondrial genome and pseudogenes. Only contigs showing a minimum of 30 reads across replicates and conditions were considered for further analysis. TOP transcripts were retrieved using their mgi gene symbol based on a gene list of known TOP-motif mRNAs curated from.^73^ All other transcripts were considered as “non-TOP”. For each contig captured in our direct RNA sequencing, the number of reads with poly(A) tails 30- to 90-nt long (referred as ∼60-nt long tails), or with poly(A) tails shorter than 30-nt long (referred as <30-nt long tails), were quantified and expressed as a percentage relative to the total number of reads obtained from the same contig. Transcripts with a p-value ≤ 0.05 were considered statistically changing their accumulation with <30-nt or ∼60-nt long poly(A) tails (Two-tailed, two sample Student’s t-test).

#### Differential transcript accumulation analysis

Differential transcript accumulation was determined using DESeq2 available as a Bioconductor package.^74^ Briefly, the DESeqDataSet file was built from the count matrix obtained from direct RNA sequencing with the DESeqDataSetFromMatrix function. The standard differential expression analysis steps (normalization, dispersion estimation and fold change estimation) were processed with the original DESeq function as described.^74^ Results tables were generated using the function results, which extracts log_2_ fold changes, Wald test *p*-values and adjusted p-values corrected for multiple testing using the Benjamini and Hochberg procedure. A significant threshold of q-value ≤ 0.05 was used for data interpretation. The overall effect of experimental covariates was visualized using the plotPCA function (Figure S7F). Comprehensive results of the DESeq2 analysis are presented in Table S1.

#### Enrichment analysis

The list of known TOP-motif mRNAs was curated from.^73^ Their enrichment among the population of transcripts accumulating with poly(A) tails <30-nt or ∼60-nt long was calculated using the hypergeometric test with k (number of successes), number of known TOP mRNA within the tested population; s (sample size), number of transcript within our tested population; M (number of successes in the population), number of known TOP mRNA available in our RNA-seq dataset; and N (population size), total number of transcripts available in our RNA-seq dataset.

#### Statistical analyses

The statistical analysis details of each experiment can be found in the corresponding figure legend. To determine whether the data met the assumptions of the statistical tests, normal distribution and equal variance across samples were tested using a Shapiro-Wilk test and a Levene test, respectively. For non-parametric comparisons between two samples, an unpaired or paired two-samples Wilcoxon test was used to consider the difference in the median value between the two distributions. For non-parametric comparisons between two or more samples, a Krustal-Wallis one-way analysis of variance applying the Bonferroni correction for multiple testing was used. When the data met the normal distribution assumption, parametric tests were used. To compare two unpaired samples with equal variance a two-samples, two-tailed Student’s t-test was used. When appropriate, the difference between paired samples was compared using a two-tailed paired Student’s t-test. To compare more than two populations with equal variance, statistical analyses were performed using a one-way ANOVA followed by Dunnett’s multiple comparison test. In case of heteroscedasticity, multiple comparisons were performed using a Games-Howell test. The two-sided Kolmogorov-Smirnov test was used to compare the cumulative distribution functions of two sample sets.

## Data Availability

• All the sequencing data are publicly available as of the date of publication and has been deposited at the Gene Expression Omnibus (GEO)^59^ with the accession numbers GSE214875 and GSE231980 also listed in the key resources table. Original Western blot and microscopy images reported in this paper are available from the lead contact upon request. • This paper does not report original code. • Any additional information required to reanalyze the data reported in this paper is available from the lead contact upon request.

## Supplemental information

Document S1. Figures S1–S7 and Tables S9 and S10

Table S1. Differential RNA accumulation analysis (DESeq2) between differentiated cells (D6) and undifferentiated cells (D0), related to Figure 1D

Table S2. Gene ontology analysis for transcripts showing differential accumulation during differentiation (from D0 to D6), related to Figure 1E Tab1 shows gene ontologies associated with downregulated transcripts. Tab2 shows gene ontologies associated with upregulated transcripts.

Table S3. Hypergeometric test for the overlap between downregulated transcripts during differentiation (from D0 to D6) and TOP mRNAs, related to Figures 1F and G Tab1 shows the mgi gene symbols for independent population of transcripts and their overlap. Tab2 shows the details of the corresponding hypergeometric test with k (number of successes), number of known TOP mRNA within the tested population; s (sample size), number of transcripts within our tested population; M (number of successes in the population), number of known TOP mRNA available in our RNA-seq dataset; and N (population size), total number of transcripts available in our RNA-seq dataset.

Table S4. Full report of Ingenuity Pathway Analysis (IPA) for the differential RNA accumulation associated with differentiation (from D0 to D6), related to Figure 2D Tab1 shows details of the canonical pathways. Tab2 reports details of the upstream analysis.

Table S5. MEME analysis, related to Figure 3C Tab1 shows a screenshot of the discovered motifs at the 5′UTR of transcripts showing an accumulation with <30-nt long tails during differentiation (from D0 to D6). Tab2 shows details for the most enriched motifs associated to Tab1. Tab3 same as Tab1, but for transcripts accumulating with ∼60-nt long tails during differentiation (from D0 to D6). Tab4 shows details for the most enriched motifs associated to Tab3. Tab5 shows a screenshot of the discovered motifs at the 3′UTR of transcripts showing an accumulation with <30-nt long tails during differentiation (from D0 to D6). Tab6 shows details for the most enriched motifs associated to Tab5. Tab7 same as Tab5, but for transcripts accumulating with ∼60-nt long tails during differentiation (from D0 to D6). Tab8 shows details for the most enriched motifs associated to Tab7.

Table S6. Hypergeometric test for the overlap between transcripts with differential poly(A) tails during differentiation (from D0 to D6) and TOP mRNAs, related to Figure 3D Tab1 shows the mgi gene symbols for independent population of transcripts and their overlap. Here, the transcripts accumulating with <30-nt long tails during differentiation (from D0 to D6) are the test population. Tab2 shows the details of the hypergeometric test corresponding to Tab1 with k (number of successes), number of known TOP mRNA within the tested population; s (sample size), number of transcripts within our tested population; M (number of successes in the population), number of known TOP mRNA available in our RNA-seq dataset; and N (population size), total number of transcripts available in our RNA-seq dataset. Tab3 same as Tab1, but here the transcripts accumulating with ∼60-nt long tails during differentiation (from D0 to D6) are the test population. Tab4 showing the details of the hypergeometric test corresponding to Tab3.

Table S7. Gene ontology analysis for transcripts with differential poly(A) tails during differentiation (from D0 to D6), related to Figure 3B Tab1 shows gene ontologies associated with transcripts accumulating with <30-nt long tails during differentiation. Tab2 same as Tab1 with transcripts depleted in tails <30-nt long during differentiation. Tab3 shows gene ontologies associated with transcripts accumulating with ∼60-nt long tails during differentiation. Tab4 same as Tab3 with transcripts depleted in tails ∼60-nt long during differentiation.

Table S8. Full report of Ingenuity Pathway Analysis (IPA) for the differential RNA translation associated with differentiation (from D0 to D6), related to Figure 4B Tab1 shows details of the canonical pathways. Tab2 reports details of the upstream analysis.
