# Supplementary material for: A unique poly(A) tail profile uncovers the stability and translational activation of TOP transcripts during neuronal differentiation
Source: iScience. 2023 Jul 27;26(9):107511. doi: 10.1016/j.isci.2023.107511 (PMC10448114; doi:10.1016/j.isci.2023.107511)
Supplement: Document S1. Figures S1–S7 and Tables S9 and S10 [file mmc1.pdf]

iScience, Volume *26*

Supplemental information

A unique poly(A) tail profile uncovers the stability and translational activation of TOP transcripts during neuronal differentiation

Marine Baptissart, Brian N. Papas, Ru-pin Alicia Chi, Yin Li, Dongwon Lee, Bhairavy Puviindran, and Marcos Morgan

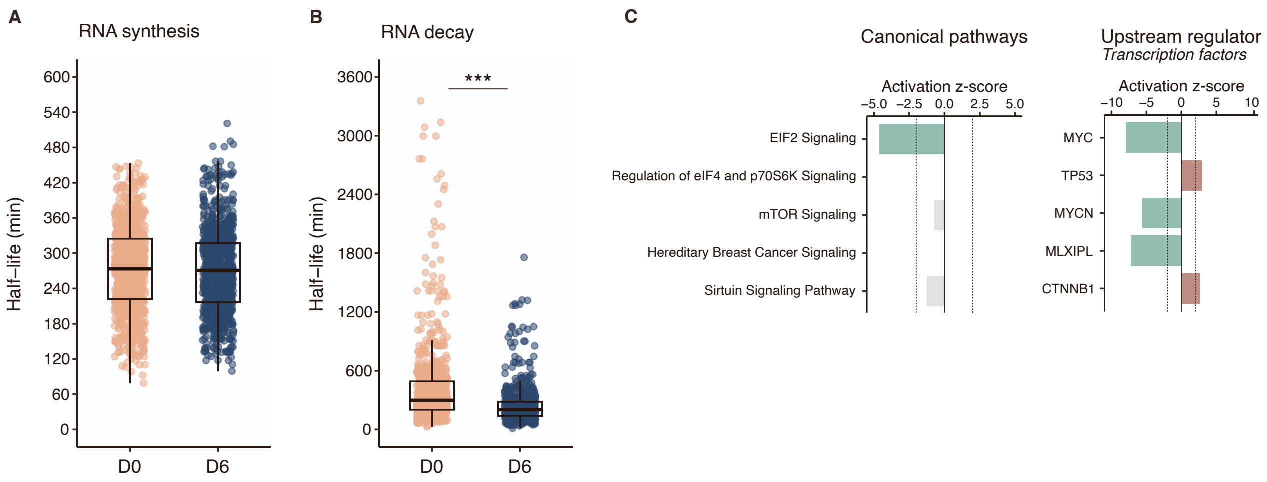

Figure S1, TOP transcripts remain highly stable through differentiation despite global mRNA decay, related to Figure 2.
(A) Boxplot of synthesis half-life for the whole transcriptome of P19 cells before differentiation (D0, orange) and after 6 days of differentiation (D6, blue). n=3 biological replicates. Paired Student's t-test, two-tailed comparing D6 to D0. (B) Boxplot as in (a) for RNA decay. n=3 biological replicates. Wilcoxon signed-rank test for paired samples comparing D6 to D0. ***$p < 0.001$. For both box plots, each dot represents an individual transcript, the center values show the medians, the boxes indicate the first and third quartiles and the bars the 10th and 90th percentiles. (C) Graphical representation of Ingenuity Pathway Analysis (IPA) for the differential RNA accumulation associated with differentiation (from D0 to D6). The top 5 Canonical pathways (left) and Upstream regulators filtered for Transcription factors (right) are ordered according to the statistical significance of their enrichment on the y-axis. The x-axis displays the predicted direction of the effect as quantified by z-scores ($z < 2$, significant negative effect in green; $z > 2$ significant positive effect in brown). eIF, Eukaryotic Initiation Factor; mTOR, Mammalian target of rapamycin; TP53, Tumor protein P53; MLXIPL, MLX interacting protein-like; CTNNB1, Catenin Beta 1.

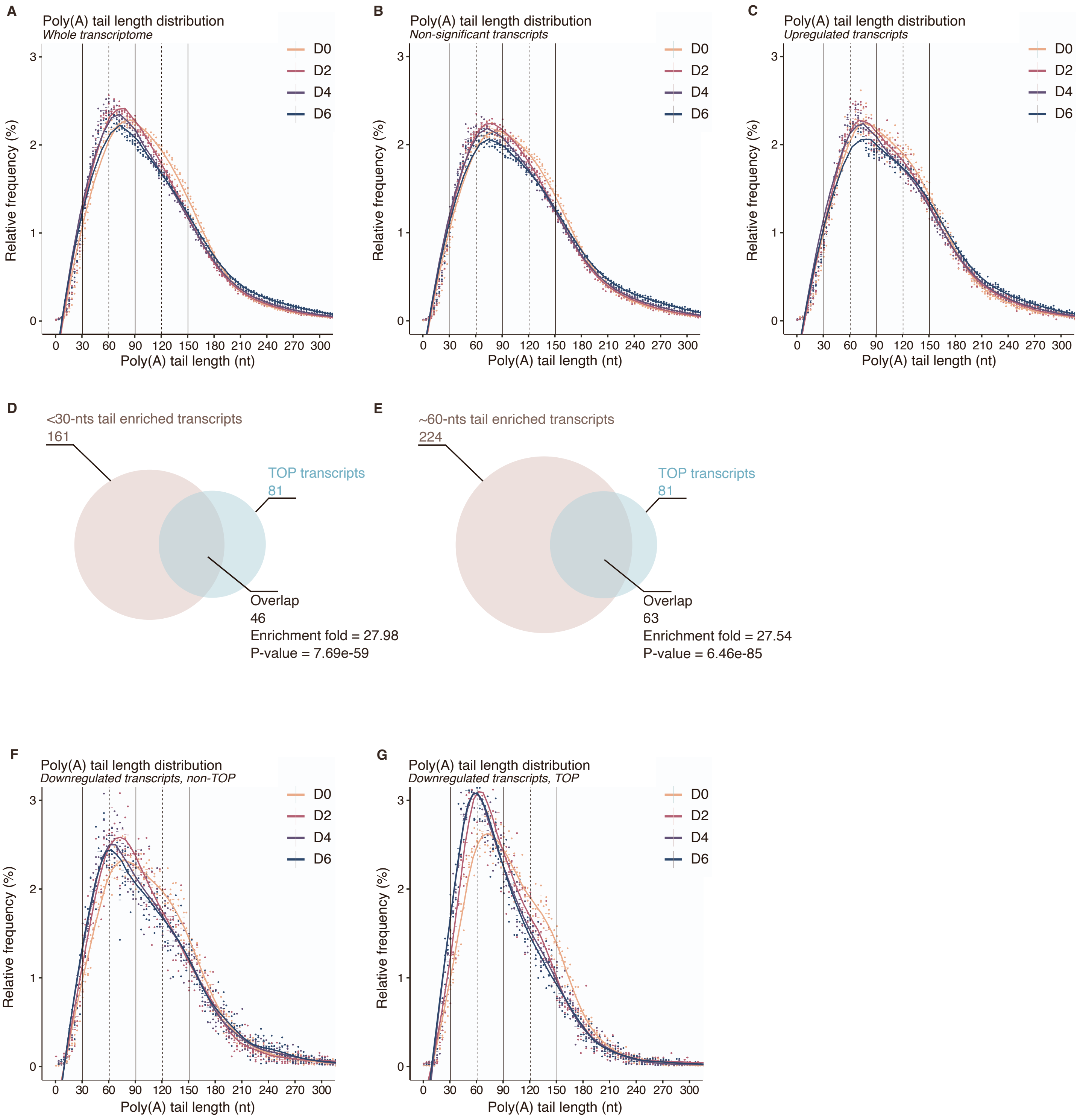

Figure S2, TOP transcripts show specific poly(A) tail-length processing during differentiation, related to Figure 3.
(A) Relative frequency plot of poly(A) tail length for the whole transcriptome before differentiation (D0, orange) and at day 2 (D2, pink), 4 (D4, purple), and 6 (D6, blue) of differentiation. (B) Relative frequency plot as in (a) for transcript with non-significant changes in RNA accumulation during differentiation (from D0 to D6). (C) Relative frequency plot as in (a) for transcript upregulated during differentiation (from D0 to D6). (D) Venn diagram showing transcripts positively enriched for <30-nts poly(A) tails (from D0 to D6) in light brown, and their intersection with TOP mRNAs in blue. Overrepresentation analysis was performed using a hypergeometric test. (E) Venn diagram as in (d) for transcripts positively enriched for ~60-nts poly(A) tails (from D0 to D6). (F) Relative frequency plot as in (a) for non-TOP downregulated transcripts (from D0 to D6). (G) Relative frequency plot as in (a) for TOP downregulated transcripts (from D0 to D6). For all plots, n=3 biological replicates. For all relative frequency plots, dots indicate values for individual replicates and the bars indicate the relative mean frequency for each poly(A) tail length.

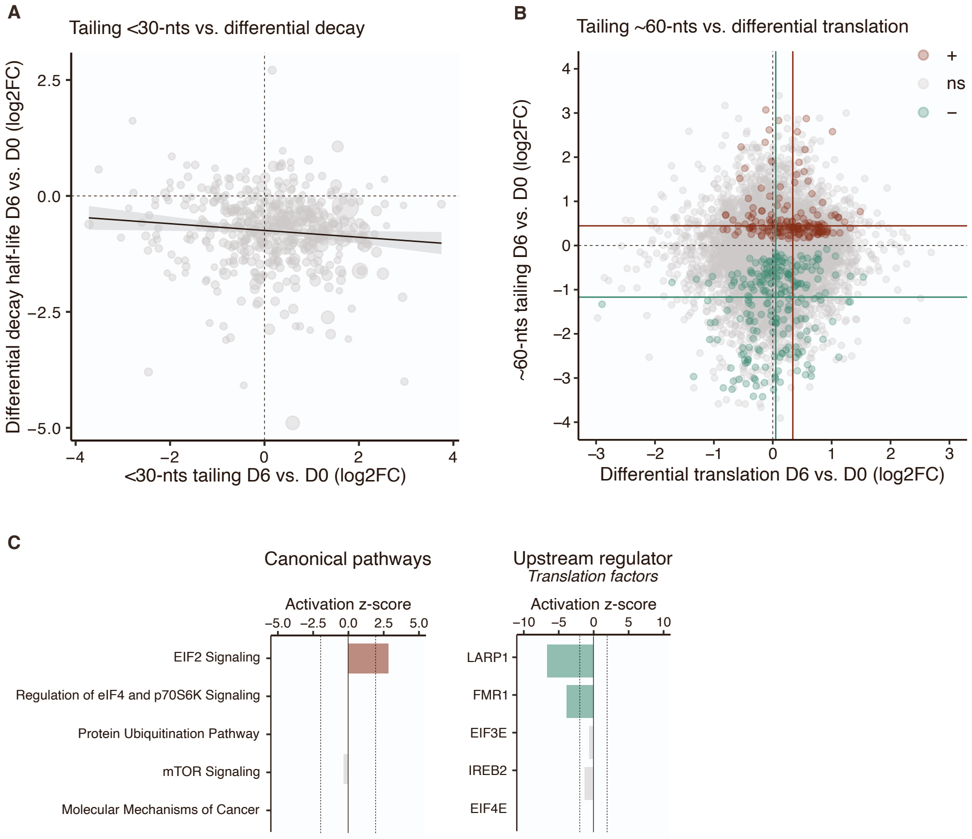

Figure S3, The poly(A) tail dynamic of TOP mRNAs is associated with their absolute stability and translational activation during neuronal differentiation, related to Figure 4.

(A) Scatter plot comparing changes in decay half-life with changes in the proportion of ~30 nucleotides (nts) long poly(A) tails of transcripts before (D0) and after differentiation (D6). n=3 biological replicates. The size of each dot is proportional to the average of the transcript relative frequency at D0 and D6. The correlation between both variables is indicated by linear regression (dark line). (B) Scatter plot comparing changes in the proportion of ~60-nts long poly(A) tails and the differential translation of transcripts during differentiation (from D0 to D6). Transcripts positively or negatively enriched for ~60-nts tails during differentiation (p-value <0.05) are indicated in brown and green, respectively. n=3 biological replicates. The medians of both variables are indicated by lines for each population of transcripts. For both scatter plots, data are representative of n=3 biological replicates. Each dot represents an individual transcript. (C) Graphical representation of Ingenuity Pathway Analysis (IPA) for the differential RNA translation associated with differentiation (from D0 to D6). The top 5 Canonical pathways (left) and Upstream regulators filtered for Translation factors (right) are ordered according to the statistical significance of their enrichment on the y-axis. The x-axis displays the predicted direction of the effect as quantified by z-scores ($z < 2$, significant predicted negative effect in green; $z > 2$, significant predicted positive effect in brown). eIF, Eukaryotic Initiation Factor; mTOR, Mammalian target of rapamycin; LARP1, La Ribonucleoprotein 1; FMR1, Fragile X Messenger Ribonucleoprotein 1; IREB2, Iron Responsive Element Binding Protein 2.

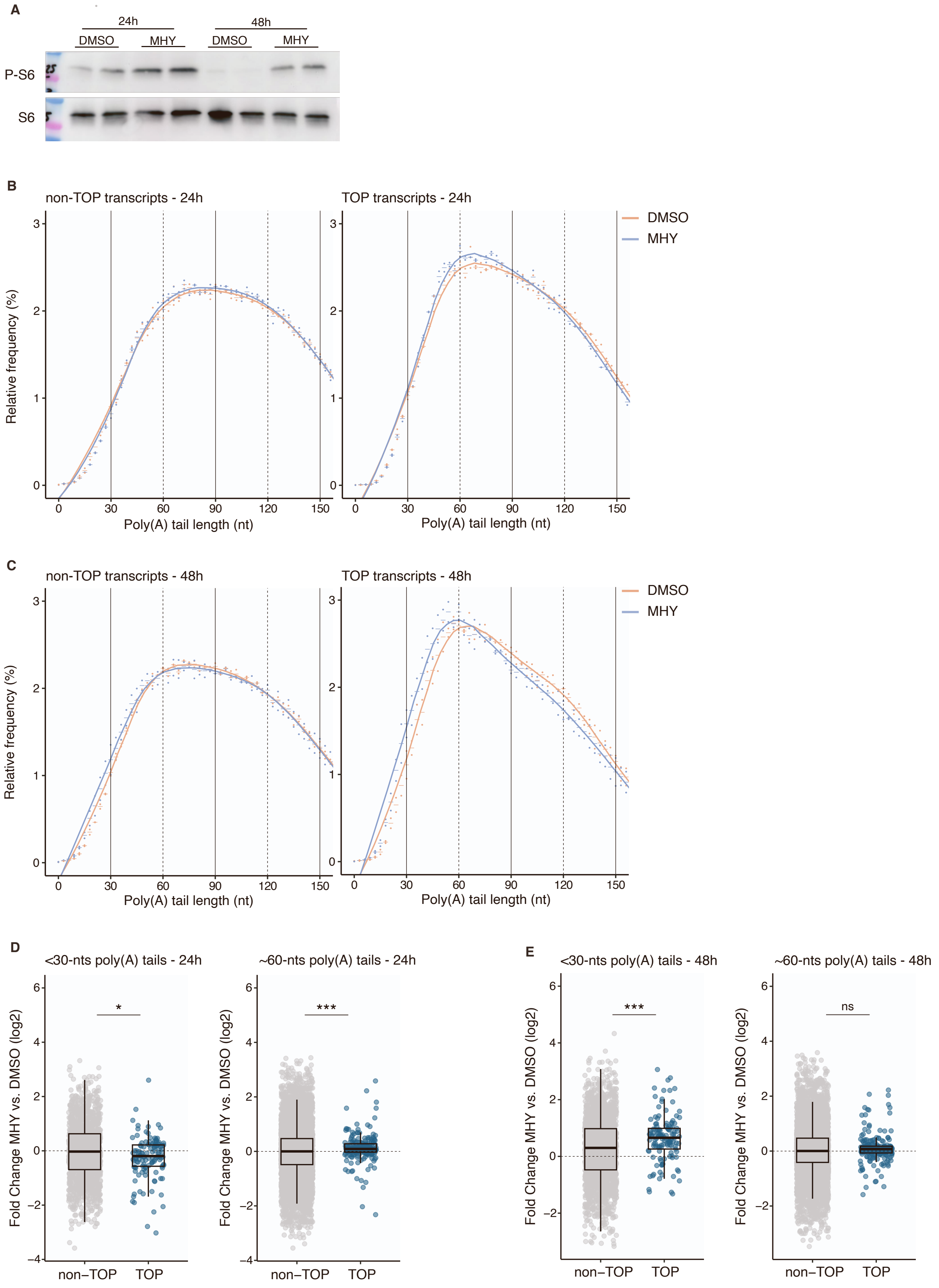

Figure S4, The mTOR/Larp1 interplay recapitulates the poly(A) dynamic of TOP mRNAs during neuronal differentiation, related to Figure 5.
(A) Representative immunoblot showing P-S6 (Ser235/236) and S6 protein accumulation in undifferentiated cells (D0) after 24 or 48hs of exposure to DMSO or MHY1485. Image representative of n=4 biological replicates, 2 independent experiments. (B) Relative frequency plot of poly(A) tail length extended to 150-nts for the non-TOP (left) and TOP (right) transcripts after 24hs of exposure to DMSO (orange) or MHY1485 (blue). n=2 biological replicates. (C) Relative frequency plot same as (B) after 48h of exposure to DMSO (orange) or MHY1485 (blue). n=2 biological replicates. For all relative frequency plots, dots indicate values for individual replicates and the bars indicate the relative mean frequency for each poly(A) tail length. (D) Box plot showing the fold change in the proportion of tails <30-nts (left) and ~60-nts (right) long 24hs after transfection with DMSO or MHY1485. TOP mRNAs are compared to non-TOP. n=2 biological replicates. Wilcoxon signed-rank test for unpaired samples comparing non-TOP to TOP transcripts. *$p < 0.05$. ***$p < 0.001$. (E) Box plot same as (D) 48h after transfection. n=2 biological replicates. Wilcoxon signed-rank test for unpaired samples comparing non-TOP to TOP transcripts. ***$p < 0.001$. ns, non significant. For all box plots, each dot represents an individual transcript, the center values show the medians, the boxes indicate the first and third quartiles and the bars the 10th and 90th percentiles.

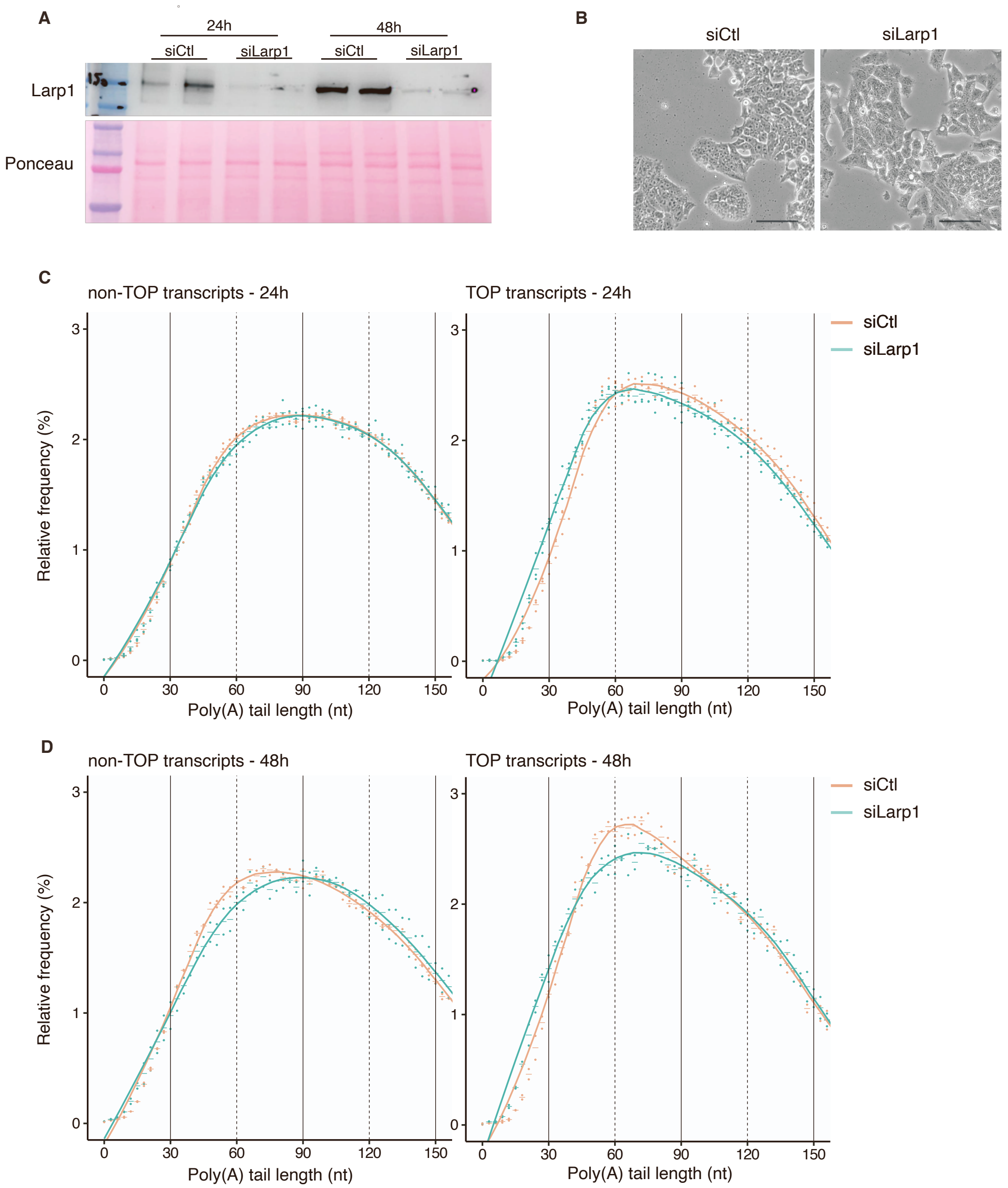

Figure S5, The mTOR/Larp1 interplay recapitulates the poly(A) dynamic of TOP mRNAs during neuronal differentiation, related to Figure 5.
(A) Representative immunoblot showing Larp1 protein accumulation in undifferentiated cells (D0) after 24 or 48h of transfection with siCtl or siLarp1. Ponceau staining of the corresponding membrane is shown as an indication of protein load. Image representative of n=4 biological replicates, 2 independent experiments. (B) Representative micrographs of P19 undifferentiated cells (D0) transfected with siCtl or siLarp1 for 48h. Scale bars: 100 μm. (C) Relative frequency plot of poly(A) tail length extended to 150-nts for the non-TOP (left) and TOP (right) transcripts after 24h of transfection with siCtl (orange) or siLarp1 (green). n=3 biological replicates. (D) Relative frequency plot as in (C) after 48h of transfection with siCtl (orange) or siLarp1 (green). n=2 biological replicates. For all relative frequency plots, dots indicate values for individual replicates and bars indicate the relative mean frequency for each poly(A) tail length.

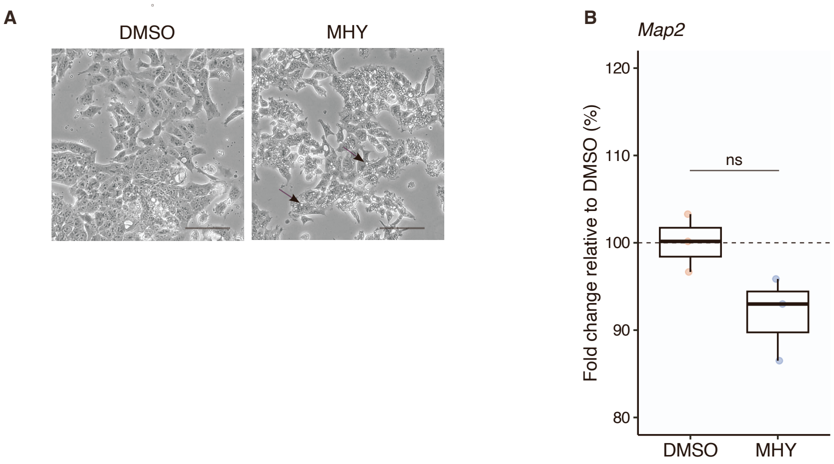

Figure S6, The timely processing of TOP mRNA poly(A) tails regulates the expansion of neuronal progenitor, related to Figure 6.
(A) Representative micrographs of undifferentiated P19 cells (D0) treated with DMSO or MHY1485 during 48h. Arrowheads indicate enlarged autophagosomes characteristic of the inhibitory effect of MHY1485 on autophagy. Scale bars: 100 μm. (B) Box plot of the relative accumulation of Map2 transcripts in response to 48h exposure to DMSO or MHY1485 measured by qPCR. n=3 biological replicates. Data are expressed relative to the DMSO condition. Each dot represents one replicate, the center values show the means, the boxes indicate the first and third quartiles and the bars indicate the 10th and 90th percentiles. Two sample Student's t-test, two-tailed comparing MHY1485 to DMSO. ns, non significant.

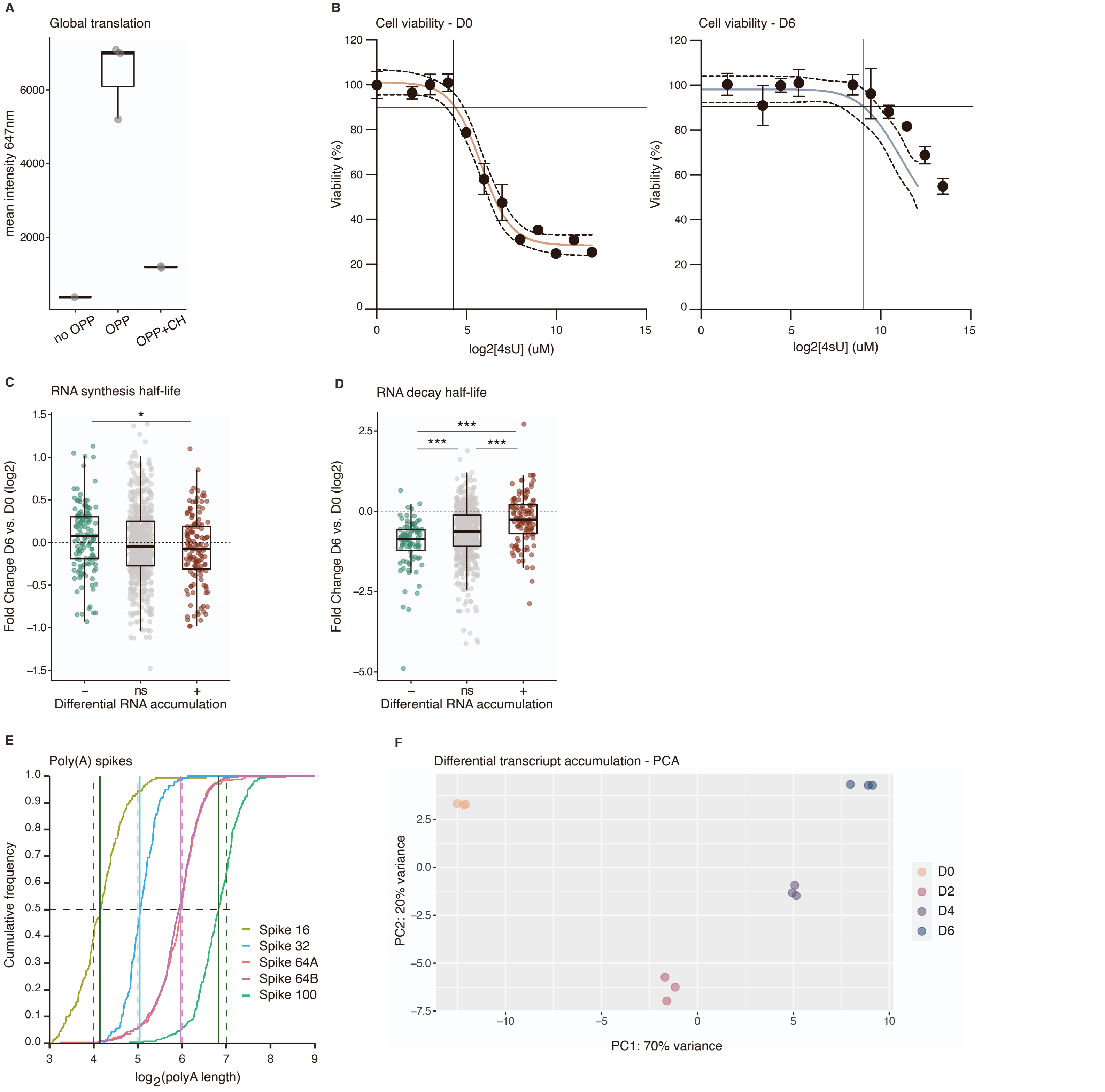

Figure S7, Calibration results for protein and RNA metabolism assays, related to Star Methods.
(A) Measure of global protein synthesis by OPP incorporation and FACS quantification in P19. The center values show the means, the boxes indicate the first and third quartiles and the bars the 10th and 90th percentiles. (B) Cell viability in response to 27 hours of exposure to increasing concentration of 4sU. Undifferentiated (day 0, D0) or differentiated (D6) cells are shown on the left or right panel, respectively. Dots indicates the relative mean value for each timepoint, the bars indicate standard deviations. The 4sU concentrations associated with 90% viability are interpolated from a sigmoidal fitness of the standard dilution. (C) Box plot showing the fold change of synthesis half-life during differentiation (D6 vs. D0) for transcripts split according to their differential accumulation (D6 vs. D0). Krustal-Wallis test corrected by Bonferroni for multiple testing. *$p < 0.05$. (D) Box plot as in (C) showing the fold change of decay half-life during differentiation (D6 vs. D0). Krustal-Wallis test corrected by Bonferroni for multiple testing. ***$p < 0.001$. (E) Cumulative frequency plot of poly(A) spike-ins relative to the poly(A) tail length (log2) measured by direct RNA sequencing. (F) PCA representation of the covariates from the differential transcript accumulation analysis comparing stages of P19 cell differentiation from D0 to D6. For all plots, n=3 biological replicates.

Oligo name	Oligo sequence
**RNA-Spike_Frw**	CGTCGAGGAGTAATACGACTCACTATAGAATCCTGGCCCAGTGAGCAA
**RNA-Spike_8**	T(8)GGCACAGTCGGCACATACACGCTCACAGGCTGATCAGCGAGCTCTA
**RNA-Spike_16**	T(16)GGCACAGTCGGCTCGTCGCGCGCACAAGGCTGATCAGCGAGCTCTA
**RNA-Spike_32**	T(32)GGCACAGTCGACAGTGCGCTGTCTATAGGCTGATCAGCGAGCTCTA
**RNA-Spike_64_a**	T(64)GGCACAGTCGTCACACTCTAGAGCGAAGGCTGATCAGCGAGCTCTA
**RNA-Spike_64_b**	GGCACAGTCGCGCTGCGAGAGACAGTAGGCTGATCAGCGAGCTCTA
**RNA-Spike_100**	GGCACAGTCGATGACAGTGCTCAGTGAGGCTGATCAGCGAGCTCTA

Table S9, Oligos used to generate spike-ins of different poly(A) tail lengths, related to Star Methods.

Barcode ID	Oligo A	Oligo B
**BC1**	/5Phos/GGCTTCTTCTTGCTCTTAGGTAGTAGGTTC	GAGGCGAGCGGTCAATTTTCCTAAGAGCAAGAAGAAGCCTTTTTTTTTT
**BC2**	/5Phos/GTGATTCTCGTCTTTCTGCGTAGTAGGTTC	GAGGCGAGCGGTCAATTTTCGCAGAAAGACGAGAATCACTTTTTTTTTT
**BC3**	/5Phos/GTACTTTTCTCTTTGCGCGGTAGTAGGTTC	GAGGCGAGCGGTCAATTTTCCGCGCAAAGAGAAAAGTACTTTTTTTTTT
**BC4**	/5Phos/GGTCTTCGCTCGGTCTTATTTAGTAGGTTC	GAGGCGAGCGGTCAATTTTAATAAGACCGAGCGAAGACCTTTTTTTTTT

Table S10, Oligos used for direct RNA library multiplexing, related to Star Methods.
